# Human Oligodendrogenic Neural Progenitor Cells Delivered with Chondroitinase ABC Facilitate Functional Repair of Chronic Spinal Cord Injury

**DOI:** 10.1016/j.stemcr.2018.10.017

**Published:** 2018-11-21

**Authors:** Satoshi Nori, Mohamad Khazaei, Christopher S. Ahuja, Kazuya Yokota, Jan-Eric Ahlfors, Yang Liu, Jian Wang, Shinsuke Shibata, Jonathon Chio, Marian H. Hettiaratchi, Tobias Führmann, Molly S. Shoichet, Michael G. Fehlings

**Affiliations:** 1 Division of Genetics and Development, Krembil Research Institute, University Health Network, 60 Leonard Avenue, Toronto, ON M5T 2S8, Canada; 2 Department of Orthopaedic Surgery, Keio University School of Medicine, 35 Shinanomachi, Shinju-ku, Tokyo 160-8582, Japan; 3 Department of Orthopaedic Surgery, Graduate School of Medical Sciences, Kyushu University, 3-1-1 Maidashi, Higashi-ku, Fukuoka 812-8582, Japan; 4 New World Laboratories Inc., 500 Boulevard Cartier Quest, Laval, QC H7V 5B7, Canada; 5 Electron Microscope Laboratory, Keio University School of Medicine, 35 Shinanomachi, Shinju-ku, Tokyo 160-8582, Japan; 6 Department of Chemical Engineering and Applied Chemistry, University of Toronto, 200 College Street, Toronto, ON M5S 3E5, Canada; 7 Department of Chemistry, University of Toronto, 80 St. George Street, Toronto, ON M5S 3H6, Canada; 8 Institute of Biomaterials & Biomedical Engineering, Terrence Donnelly Centre for Cellular and Biomolecular Research, University of Toronto, 160 College Street, Toronto, ON M5S 3E1, Canada; 9 Institute of Medical Sciences, University of Toronto, 1 King's College Circle, Toronto, ON M5S 1A8, Canada; 10 Department of Surgery and Spinal Program, University of Toronto, 1 King's College Circle, Toronto, ON M5S 1A8, Canada; 11 Department of Surgery, Division of Anatomy, Donnelly Centre, University of Toronto, 160 College Street, Toronto, ON M5S 3E1, Canada

**Keywords:** neural progenitor cells, oligodendrocyte, chronic spinal cord injury, glial scar, ChABC, cell transplantation, combinatorial therapy, remyelination, crosslinked methylcellulose hydrogel, regenerative medicine

## Abstract

Treatment of chronic spinal cord injury (SCI) is challenging due to cell loss, cyst formation, and the glial scar. Previously, we reported on the therapeutic potential of a neural progenitor cell (NPC) and chondroitinase ABC (ChABC) combinatorial therapy for chronic SCI. However, the source of NPCs and delivery system required for ChABC remained barriers to clinical application. Here, we investigated directly reprogrammed human NPCs biased toward an oligodendrogenic fate (oNPCs) in combination with sustained delivery of ChABC using an innovative affinity release strategy in a crosslinked methylcellulose biomaterial for the treatment of chronic SCI in an immunodeficient rat model. This combinatorial therapy increased long-term survival of oNPCs around the lesion epicenter, facilitated greater oligodendrocyte differentiation, remyelination of the spared axons by engrafted oNPCs, enhanced synaptic connectivity with anterior horn cells and neurobehavioral recovery. This combinatorial therapy is a promising strategy to regenerate the chronically injured spinal cord.

## Highlights

• Sustained biomaterial delivery of ChABC successfully degraded CSPGs
• XMC-ChABC promoted differentiation of oNPCs to more oligodendrocytes
• XMC-ChABC increased the long-term survival and integration of grafted oNPCs
• XMC-ChABC and oNPC combinatorial therapy is a promising treatment for chronic SCI

## Graphical Abstract

**Figure:**
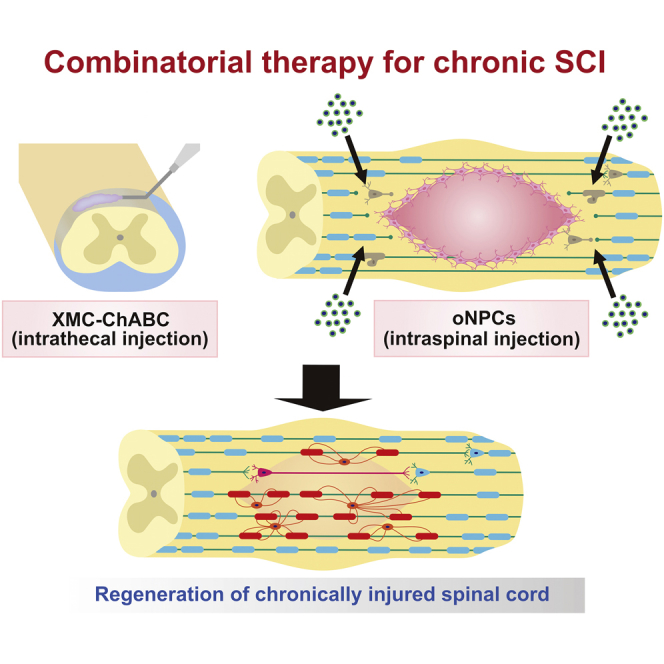

## Teaser

Fehlings and colleagues investigated the use of directly reprogrammed human neural progenitor cells biased toward an oligodendrogenic fate (oNPCs) combined with sustained delivery of chondroitinase ABC to treat chronic spinal cord injury in an immunodeficient rat model. This combinatorial therapy increased long-term survival of oNPCs, facilitated greater oligodendrocyte differentiation, remyelination of the spared axons by engrafted oNPCs, and neurobehavioral recovery.

## Introduction

Neural progenitor cells (NPCs) represent a promising regenerative strategy to target several CNS disorders (Lindvall and Kokaia, 2006). Neurobehavioral recovery following NPC transplantation into the injured spinal cord has been reported in both rodent and non-human primate models (Cummings et al., 2005, Iwanami et al., 2005, Karimi-Abdolrezaee et al., 2010). Although NPCs have therapeutic value, their translational potential is hindered by limited availability, immunologic complications, and ethical concerns. Recently, transplantation of induced pluripotent stem cell (iPSC)-derived NPCs and iPSC-derived oligodendrocyte progenitor cell-enriched NPCs has demonstrated therapeutic promise (Kawabata et al., 2016, Nori et al., 2011, Suzuki et al., 2017). However, previous studies have identified the potential for tumorigenicity, immunogenicity, and genetic and epigenetic abnormalities following iPSC-based cell transplantation (Nori et al., 2015). To avoid the risks associated with NPCs and iPSCs, we focused on human directly reprogrammed NPCs (drNPCs), which were directly generated from somatic cells, thus avoiding the pluripotent state (Nagoshi et al., 2018).

Within the injured spinal cord environment, oligodendrocytes are highly susceptible to the cytotoxic conditions found both local and distant to the lesion epicenter, leading to demyelination of preserved axons (Dong et al., 2003). James et al. (2011) investigated the temporal pattern of conduction failure in individual fibers across a contusion injury and examined changes in their conduction properties from acute to chronic stages of injury. Acutely (1–7 days) after spinal cord injury (SCI), complete conduction block was observed in ascending dorsal column axons, followed by a period of improved conduction during the subacute (2–4 weeks) phase, without further improvement at the chronic (3–6 months) phase. At 6 months after SCI, 16% of sampled fibers were capable of conducting across the lesion. Furthermore, this study demonstrated a population of axons (20% of the fibers tested) which are chronically demyelinated and viable but unable to conduct under normal physiological conditions (James et al., 2011). Using detailed histological analyses, Totoiu and Keirstead (2005) observed that the number of demyelinated axons progressively increased up to 450 days after injury. Although remyelination of axons was observed from 14 to 450 days post-SCI, it was found to be incomplete. The results of the previous studies indicated that chronic and progressive demyelination represents an important target for cell transplantation therapy. Previous studies have revealed the biological importance of remyelination and tissue sparing by graft-derived cells for neurobehavioral recovery following the transplantation of NPCs into the injured spinal cord (Hawryluk et al., 2014, Yasuda et al., 2011). Thus, remyelinating spared axons and promotion of neural plasticity and/or tissue sparing by graft-derived oligodendrocytes represents a promising therapeutic strategy following SCI (Trounson and McDonald, 2015). Building on these findings, we recently developed a novel method to generate human iPSC-derived NPCs or drNPCs biased toward an oligodendrogenic fate (oNPCs) for the treatment of SCI (Khazaei et al., 2017, Nagoshi et al., 2018). Transplantation of oNPCs in the subacute phase of SCI showed greater differentiation into oligodendrocytes, axonal remyelination, and tissue sparing, which ultimately resulted in the recovery of motor function (Nagoshi et al., 2018).

The majority of studies have reported that NPCs transplanted in the subacute period following SCI have therapeutic effects (Cummings et al., 2005, Iwanami et al., 2005). Unfortunately, most patients with SCI have progressed past the subacute period, and effective therapies to target the chronic phase of SCI are greatly lacking (Nishimura et al., 2013, Suzuki et al., 2017). Although a number of animal model studies have aimed to achieve neurobehavioral recovery in the chronic phase of SCI with NPC transplantation, functional recovery has not been observed (Cusimano et al., 2012, Parr et al., 2007). Due to the inhibitory microenvironment of the chronically injured spinal cord, which includes a cystic cavity with surrounding glial scar as well as neuronal and glial cellular loss, repair and regeneration of the injured cord has proven challenging (Silver and Miller, 2004). The glial scar consists of a non-neural lesion core (fibrotic scar) and an astrocytic scar border (Sofroniew, 2018). The non-neural lesion core is comprised of stromal cells and extracellular matrix molecules including fibronectin, collagen, proteoglycans, and laminin (O'Shea et al., 2017). The astrocyte scar forms a border between the non-neural lesion core and adjacent spared neural tissue and restricts the spread of inflammation (Burda and Sofroniew, 2014). After SCI, a phenotypic change to astrocytes occurs, known as reactive astrogliosis. Naive astrocytes undergo a change including increased glial fibrillary acidic protein (GFAP) expression, hypertrophy, and process extension, resulting in a characteristic phenotype of reactive astrocytes within several days after SCI (Hara et al., 2017, Okada et al., 2006). Subsequently, reactive astrocytes overlap their processes and transform into scar-forming astrocytes (Hara et al., 2017, Silver and Miller, 2004). In SCI lesions, chondroitin sulfate proteoglycans (CSPGs) are produced not only by astrocytes, but also pericytes, fibroblast lineage cells, and inflammatory cells. CSPGs comprise the main inhibitory component of the glial scar (Anderson et al., 2016, Sofroniew, 2018). Importantly, a recent study demonstrated that genetically targeted astrocyte ablation did not reduce CSPGs, suggesting that astrocytes are not the primary producers of CSPGs in SCI lesions (Anderson et al., 2016). The inhibitory CSPGs limit integration and migration of grafted cells, which prevents remyelination of the spared axons and regeneration of neural circuits (Grijalva et al., 1996, Nishimura et al., 2013). To counter the CSPGs, the enzyme chondroitinase ABC (ChABC) has been shown to degrade the sulfated glycosaminoglycan chains on the CSPGs (Moon et al., 2001) and promote neural plasticity and functional recovery after acute SCI (Bradbury et al., 2002). Moreover, we have previously shown that a combinatorial therapy of ChABC and NPCs or iPSC-derived NPCs increased the long-term survival of NPCs and optimized their integration and migration in chronic SCI, resulting in neurobehavioral recovery (Fuhrmann et al., 2018, Karimi-Abdolrezaee et al., 2010, Suzuki et al., 2017).

One of the challenges of providing sustained delivery of ChABC is the thermal instability of the enzyme (Tester et al., 2007), which limits the activity to less than 4 days *in vivo* (Crespo et al., 2007). In previous SCI studies, ChABC has been delivered continuously using an osmotic mini-pump with intrathecal catheters (Karimi-Abdolrezaee et al., 2010, Suzuki et al., 2017). However, the mini-pump delivery system is prone to dislodgement, can be associated with off-target delivery, is invasive, and is associated with complications such as infection and arachnoiditis (Follett et al., 2004). Previously, we demonstrated that an affinity-based release of ChABC from a crosslinked methylcellulose (XMC) hydrogel could reduce CSPG levels at the injury site *in vivo* for 2 weeks and promote functional repair following a single intrathecal injection (Pakulska et al., 2013, Pakulska et al., 2017). To achieve affinity release, ChABC is delivered as a fusion protein with Src homology 3 (SH3-ChABC), and XMC is modified with an SH3 binding peptide.

The present study aimed to assess the efficacy of a combinatorial strategy employing an XMC hydrogel containing ChABC and human directly reprogrammed oNPCs to treat chronic SCI. Here, in a clip-contusion chronic SCI model using immunodeficient rats, we degraded CSPGs with a single intrathecal injection of an innovative XMC hydrogel containing ChABC. Next, we transplanted clinically relevant oNPCs 1 week after the intrathecal injection to assess the therapeutic potential of this combinatorial therapy in the chronically injured spinal cord.

## Results

### The Effect of Injury-Induced CSPGs on oNPC Differentiation *In Vitro*

**Figure 1:**
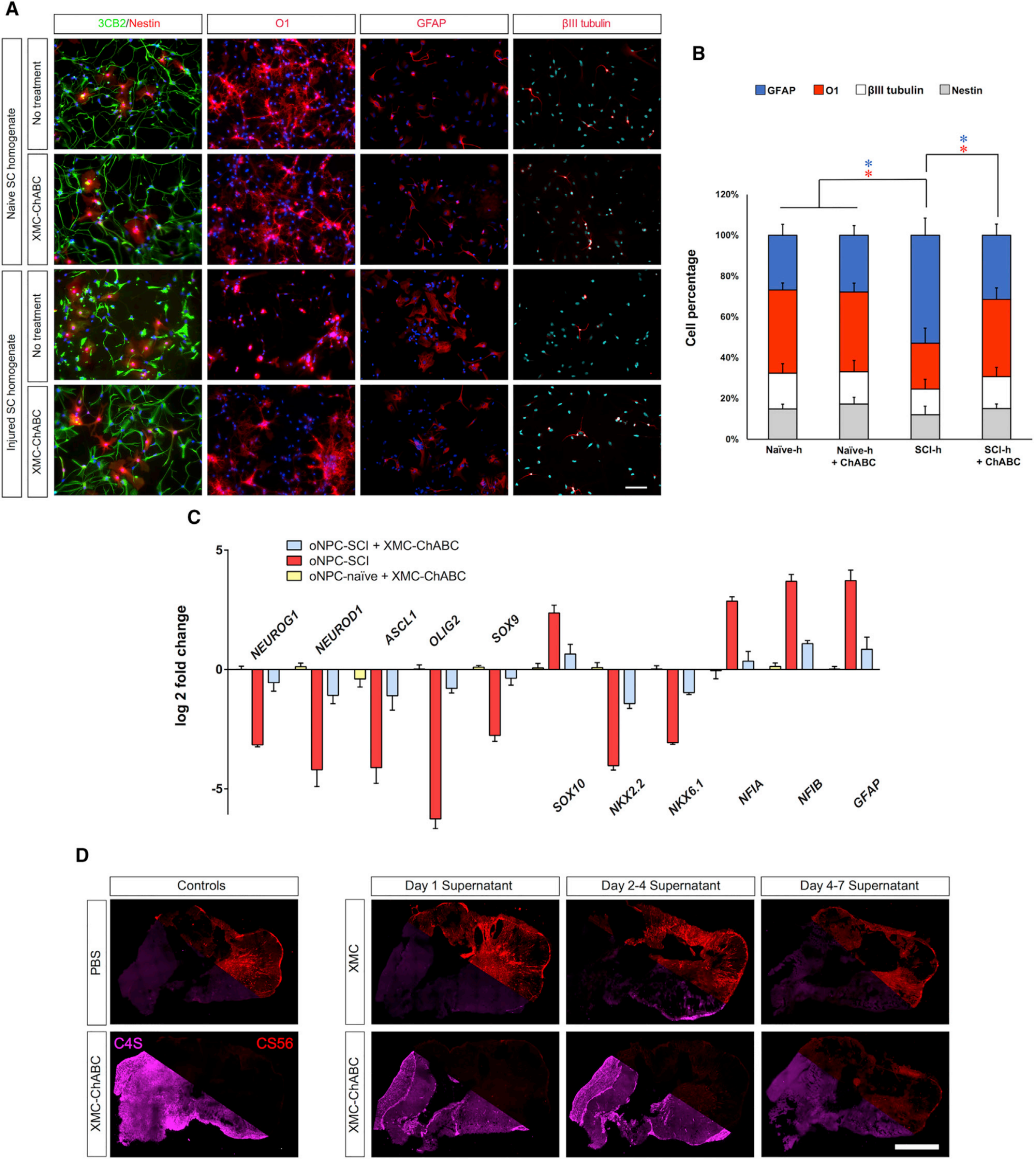
*In Vitro* oNPC Differentiation Assay with or without CSPGs oNPCs cultured on dishes coated with spinal cord homogenates from uninjured (Naive-h) or SCI-lesioned animals (SCI-h) were treated with or without XMC-ChABC for a week. (A) Cells were fixed and stained for the neural progenitor cell marker (Nestin, red), radial glial cell marker (3CB2, green), oligodendrocyte marker (O1, red), astrocyte marker (GFAP, red), or neuronal marker (βIII-tubulin, red). Scale bar, 30 μm. (B) The percentage of cells positive for GFAP, O1, βIII-tubulin, or Nestin was quantified (n = 3 biological replicates/group). Values are expressed as the means ± SD. ^∗^p < 0.05. (C) qRT-PCR analysis of the expression profile of neurogenic, astrocytogenic, and oligodendrogenic transcription factors in oNPCs cultured on SCI-h after treatment with or without XMC-ChABC relative to control-oNPCs cultured on Naive-h with no treatment. Data represent the mean log2-fold change in gene expression relative to control cells (n = 3 biological replicates/group). Values are expressed as the means ± SEM. (D) Representative images of CS56 CSPG and C4S stub immunohistochemistry of injured rodent spinal cord sections treated for 4 hr with PBS (negative control), stock ChABC enzyme (positive control), or incubated supernatant from XMC or XMC-ChABC biomaterial. XMC-ChABC supernatant incubated during day 1, days 2–4, or days 5–7 all degraded *ex vivo* CSPGs while control XMC supernatant did not.

We sought to determine if treatment with a XMC hydrogel containing ChABC (XMC-ChABC) could counter the preferential differentiation bias of oNPCs to astrocytes that is typically observed in the injured spinal cord niche. oNPCs were cultured in the absence of fibroblast growth factor 2 (FGF2)/epidermal growth factor (EGF) on coverslips coated with 100 μg/mL homogenate from the injured (SCI-h) or naive spinal cord (Naive-h) for 1 week. Withdrawal of FGF2/EGF for this period resulted in the advancement of the majority of cells to radial glial cells expressing 3CB2, while around 15% of cells remained in the neural progenitor stage, as demonstrated by Nestin expression, after 1 week of treatment. Culturing oNPCs in SCI-h resulted in a significant increase in the number of GFAP^+^ cells (52.9% ± 8.4%) compared with cells cultured in Naive-h (26.8% ± 5.3%; p < 0.05). A significant decrease in the number of cells expressing the oligodendrocyte marker O1 was observed when cultured with SCI-h (22.5% ± 7.3%) compared with cells cultured in Naive-h (40.8% ± 3.4%; p < 0.05) (Figures 1A and 1B). This effect was also confirmed using other oligodendrocyte markers including CNPase, O4 and myelin basic protein (MBP) (Figure S1). However, no significant change in the number of β-tubulin isotype III (βIII-tubulin)-positive neurons was observed in SCI-h cells (12.5% ± 4.8%) compared with cells treated with Naive-h (17.5% ± 4.6%) (Figures 1A and 1B). Treatment with XMC-ChABC mitigated this effect, with astrocyte differentiation (GFAP^+^ cells) in the SCI-h condition (31.4% ± 5.4%) maintained at a level similar to cells that were cultured on Naive-h (26.8% ± 5.3%) (Figures 1A and 1B).

Furthermore, the expression of transcription factors (TFs) was influenced by SCI-h. For oNPCs cultured with SCI-h, the expression of pro-astrocytic TFs (*NFIA* and *NFIB*) was highly upregulated compared with control cells cultured on Naive-h. Conversely, the expression of pro-neuronal TFs (*NEUROG1*, *NEUROD1*, and *ASCL1*) and pro-oligodendrocytic TFs (*OLIG2*, *SOX9*, *NKX2*.*2*, and *NKX6*.*1*) were highly downregulated compared with control cells cultured on Naive-h. Notably, however, treatment with XMC-ChABC resulted in downregulation of the expression of pro-astrocytic TFs (*NFIA* and *NFIB*) and upregulation of both pro-neuronal and pro-oligodendrocytic TFs. This experiment provides direct evidence that CSPGs produced in the post-SCI microenvironment influence the differentiation of transplanted cells and bias their differentiation toward astrocytes (Figure 1C).

### XMC-ChABC Degrades Chronic Scar CSPGs

**Figure 2:**
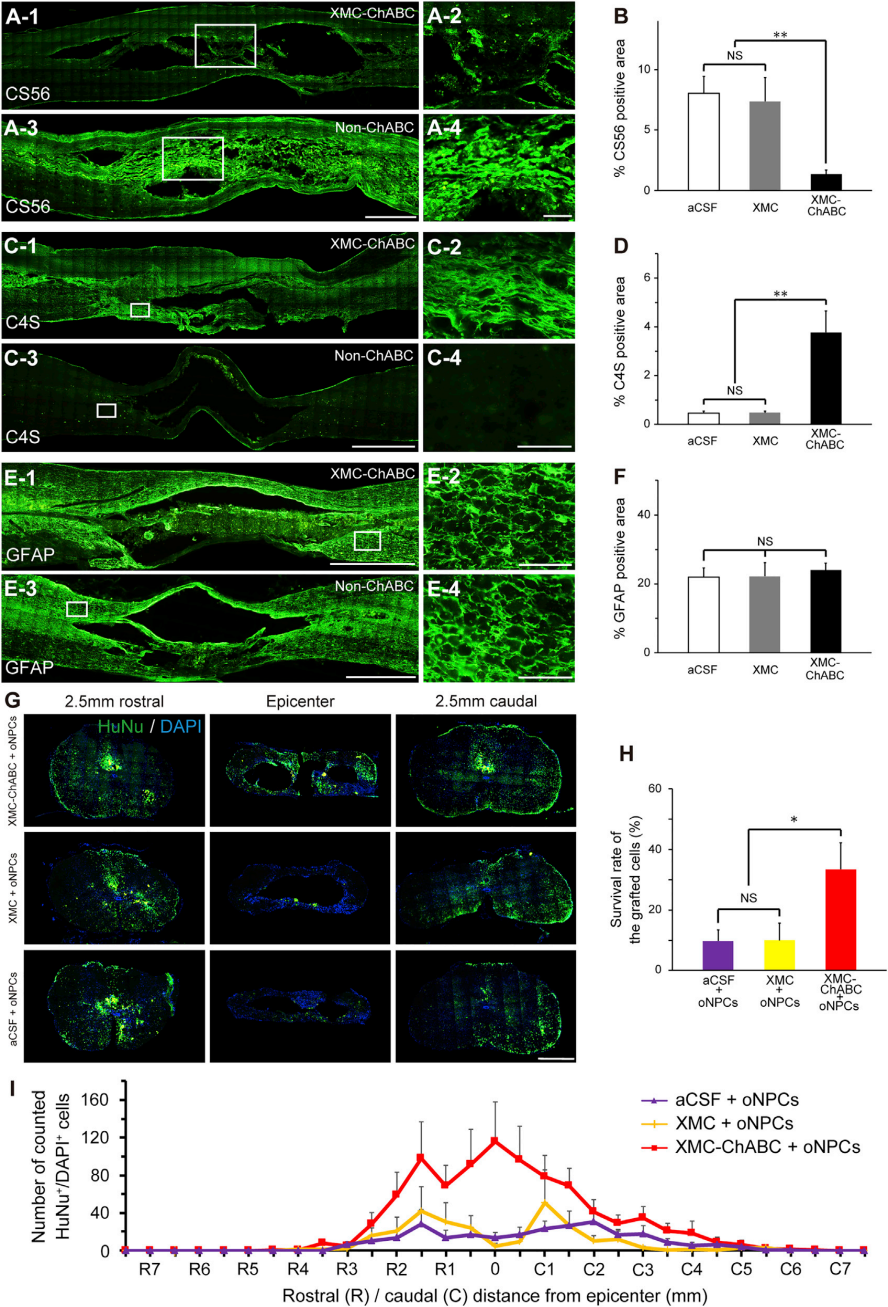
Successful Degradation of CSPGs by XMC-ChABC Treatment Enhances the Survival of Grafted oNPCs (A) Representative images of CS56^+^ CSPGs. A-2 and A-4: boxed area in A-1 and A-3, respectively. (B) CS56^+^ area (%) at 7 weeks after SCI (XMC-ChABC, n = 10; XMC, n = 6; aCSF, n = 8). (C) Representative images of C4S + degradant of CSPGs. C-2 and C-4: boxed area in C-1 and C-3, respectively. (D) C4S^+^ area (%) at 7 weeks after SCI (XMC-ChABC, n = 10; XMC, n = 6; aCSF, n = 8). (E) Representative images of GFAP^+^ astrocytes. E-2 and E-4: boxed area in E-1 and E-3, respectively. (F) GFAP^+^ area (%) at 7 weeks after SCI (XMC-ChABC, n = 10; XMC, n = 6; aCSF, n = 8). (G) Representative images of HuNu^+^/DAPI^+^-grafted oNPCs. (H) Survival rate of the grafted oNPCs at 19 weeks after SCI (n = 7/group). (I) HuNu^+^/DAPI^+^ cell distribution at 19 weeks after SCI (n = 7/group). Values are expressed as the means ± SEM. ^∗^p < 0.05, ^∗∗^p < 0.01. NS, non-significant. Scale bars, 1,000 μm (A-3, C-3, E-1, and E-3), 500 μm (G), 200 μm (A-4), and 100 μm (C-4, E-2, and E-4).

When lightly fixed *ex-vivo*-injured rodent spinal cord cryosections are incubated with XMC-ChABC supernatant, chondroitin sulfate (CS56) and chondroitin-4-sulfate (C4S) immunohistochemistry demonstrates rapid degradation of long-chain CSPGs. This effect is preserved when the media are obtained from day 1, day 2 to 4, and day 5 to 7 incubations. When XMC or PBS alone are used, the effect is not observed (Figure 1D). T cell-deficient athymic Rowett nude (RNU) rats were used for the xenograft experiments. After laminectomy, a clinically relevant clip-contusion SCI was induced at thoracic level 7 (Th7). Intrathecal injection of XMC-ChABC was performed 6 weeks after injury. Injection of XMC-ChABC resulted in a significant reduction in anti-CS56^+^ CSPGs in the perilesional area (1.4% ± 0.3%) as compared to artificial cerebrospinal fluid (aCSF) treatment (8.0% ± 1.4%; p < 0.01) and XMC hydrogel without ChABC treatment (7.4% ± 1.9%; p < 0.01) at 7 weeks after SCI (1 week after injection) (Figures 2A and 2B). Immunohistochemistry performed with anti-C4S, which detects the tetrasaccharide linker region, further confirmed the successful degradation of CSPGs in the XMC-ChABC group at 7 weeks post-SCI. While XMC-ChABC-treated animals showed an expression of C4S (3.8% ± 0.9%), no apparent endogenous C4S immunoreactivity was observed in the aCSF (0.5% ± 0.2%; p < 0.01) or XMC (0.5% ± 0.1%; p < 0.01) groups (Figures 2C and 2D). Next, we examined the effect of XMC-ChABC on the population of reactive astrocytes in the glial scar around the SCI lesion. There was no significant difference in the expression of GFAP between XMC-ChABC and non-ChABC groups at 7 weeks after injury (Figures 2E and 2F).

### XMC-ChABC Treatment Promotes Long-Term Survival of oNPCs in Chronic SCI

At 1 week after intrathecal injection (7 weeks after SCI), we performed an intraspinal injection of oNPCs (4 × 10^5^ cells, 8 μL) or aCSF (8 μL). To investigate the effects of CSPG degradation on oNPC survival in chronic SCI, we compared the survival rate of the grafted cells at 12 weeks after transplantation in combination with intrathecal injection of XMC-ChABC, XMC, or aCSF. We observed a significant increase in the number of surviving oNPCs in the XMC-ChABC group (33.4% ± 8.8%) compared with the non-ChABC groups (10.0% ± 5.6% in the XMC group and 9.7% ± 3.7% in the aCSF group; p < 0.05) (Figures 2G and 2H). Although the grafted cells in the XMC-ChABC group strongly integrated around the lesion epicenter, non-ChABC groups showed less cell integration (Figures 2G and 2I).

### oNPCs Differentiate Preferentially toward an Oligodendroglial Lineage with XMC-ChABC Treatment

**Figure 3:**
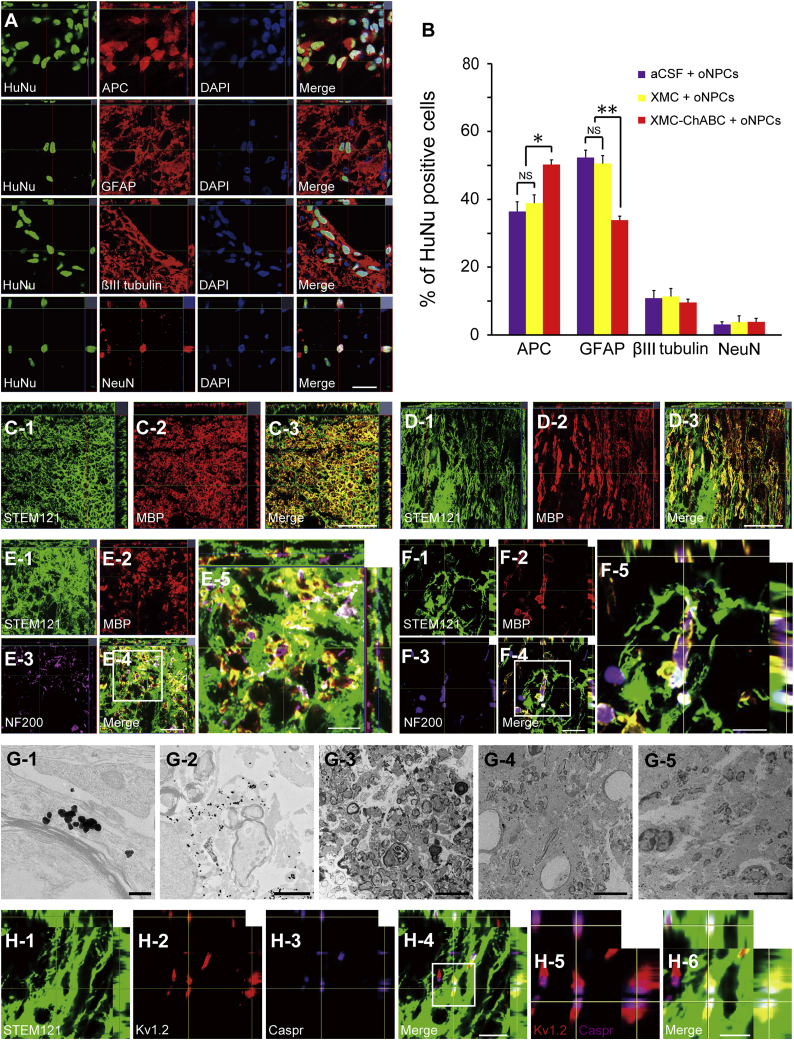
Grafted oNPCs Mainly Differentiate into Oligodendrocytes and Contribute to Remyelination of Spared Axons (A) Representative images of HuNu^+^ grafted cells immunostained for the markers adenomatous polyposis coli (APC) (oligodendrocytes), GFAP (astrocytes), βIII-tubulin (all neurons), and NeuN (neurons). (B) Percentages of cell-type-specific marker-positive cells among HuNu^+^ grafted cells at 19 weeks after SCI (n = 5/group). (C and D) Representative images of axial (C) and sagittal (D) sections stained for STEM121 and MBP. STEM121^+^/MBP^+^ oNPC-derived mature oligodendrocytes were observed. (E and F) Representative images of axial (E) and sagittal (F) sections stained for STEM121, MBP, and NF200. E-5 and F-5: boxed area in E-4 and F-4, respectively. Many STEM121^+^/MBP^+^ graft-derived myelin sheaths were observed around NF200^+^ axons. (G) Representative immunoelectron microscopic images from the XMC-ChABC + oNPCs (G-1, G-2, and G-3), aCSF + oNPCs (G-4), and XMC + oNPCs (G-5) groups. Grafted cells were detected by the black dots observed upon STEM121 staining. STEM121^+^ black dots were often observed in the outer cytoplasm of the myelin sheath (G-1 and G-2). There were more remyelinated axons surrounded by STEM121^+^ grafted cells in the XMC-ChABC + oNPCs group (G-3) than in aCSF + oNPCs (G-4) and XMC + oNPCs (G-5) groups. (H) Representative images of sagittal sections stained for STEM121, Kv1.2, and Caspr in XMC-ChABC + oNPCs group. Kv1.2^+^ juxtaparanodal voltage-gated potassium channel and Caspr^+^ paranodal protein identified nodes of Ranvier (H-5). Nodes of Ranvier were observed in STEM121^+^ myelin sheathes (H-4 and H-6). H-5 and H-6: boxed area in H-4. Values are expressed as the means ± SEM. ^∗^p < 0.05, ^∗∗^p < 0.01. NS, non-significant. Scale bars, 50 μm (A, C-3, D-3, and E-4), 20 μm (E-5 and F-4), 10 μm (F-5 and H-4), 5 μm (H-6, G-3, G-4, and G-5); 2 μm (G-2), and 200 nm (G-1).

Histological analyses were performed 12 weeks after transplantation. Grafted oNPCs differentiated into adenomatous polyposis coli CC-1^+^ oligodendrocytes, GFAP^+^ astrocytes, as well as neuronal nuclei (NeuN)^+^ and β-tubulin isotype III (βIII-tubulin)^+^ neurons (Figure 3A). oNPCs in the XMC-ChABC group demonstrated a higher proportion of cells differentiating to oligodendrocytes (50.2% ± 1.4%) compared with cells in the non-ChABC groups (38.8% ± 2.5% in the XMC group and 36.4% ± 2.8% in the aCSF group; p < 0.05). Further, cells in the XMC-ChABC group differentiated to astrocytes less frequently (33.8% ± 1.2%) compared with non-ChABC groups (50.5% ± 2.3% in the XMC group and 52.3% ± 2.1% in the aCSF group; p < 0.01). There was no significant difference in βIII-tubulin^+^ (9.6% ± 0.9% in the XMC-ChABC group, 11.3% ± 2.3% in the XMC group, and 10.8% ± 2.3% in the aCSF group) and NeuN^+^ (3.9% ± 0.9% in the XMC-ChABC group, 3.8% ± 1.9% in the XMC group, and 3.1% ± 0.9% in the aCSF group) neurons (Figure 3B).

### Grafted oNPC-Derived Mature Oligodendrocytes Contribute to Remyelination

**Figure 4:**
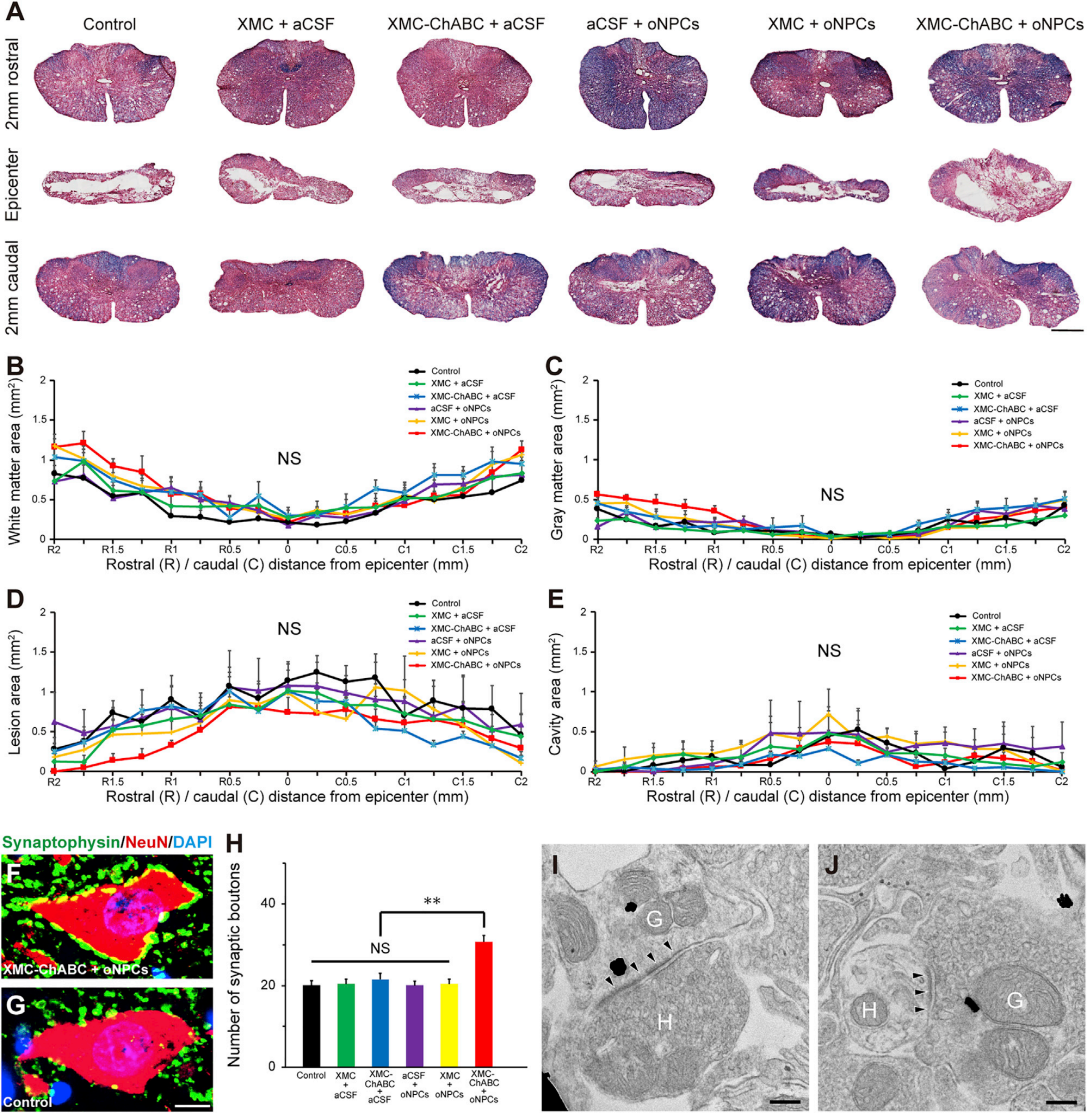
The XMC-ChABC and oNPCs Combinatorial Therapy Did Not Promote Tissue Preservation but Did Promote Enhanced Synaptic Contacts with Anterior Horn Neurons after Chronic SCI (A) Representative Luxol fast blue- and H&E-stained images of the spinal cord at the lesion epicenter and 2 mm rostral and caudal from the epicenter in control, XMC + aCSF, XMC-ChABC + aCSF, aCSF + oNPCs, XMC + oNPCs, and XMC-ChABC + oNPCs groups. (B) Quantitative analysis of the white matter area between 2 mm rostral and caudal from the epicenter (n = 5/group). (C) Quantitative analysis of the gray matter area (n = 5/group). (D) Quantitative analysis of the lesion area (n = 5/group). (E) Quantitative analysis of the cavity area (n = 5/group). (F) Representative images of synaptic contacts with the anterior horn neurons of the XMC-ChABC + oNPCs group stained for synaptophysin and NeuN. (G) Representative images of synaptic connections in the anterior horn neurons of the control group. (H) Quantitative analysis of the synaptophysin^+^ presynaptic boutons in the anterior horn neurons at 5–7 mm caudal to the lesion epicenter (n = 50 neurons; five rats per group). (I and J) Immunoelectron microscopy images showed synapse formation between host rat neurons and graft-derived STEM121^+^ (black dots) human neurons in the XMC-ChABC + oNPCs group caudal to the lesion epicenter. Presynaptic and postsynaptic structures indicated transmission from a host neuron to a graft-derived neuron (I), and from a graft-derived neuron to a host neuron (J). (H) Host neuron and (G) graft-derived neuron. Arrowheads indicate postsynaptic density. Values are expressed as the means ± SEM. ^∗∗^p < 0.01. NS, non-significant. Scale bars, 500 μm (A), 10 μm (G), and 200 nm (I and J).

**Figure 5:**
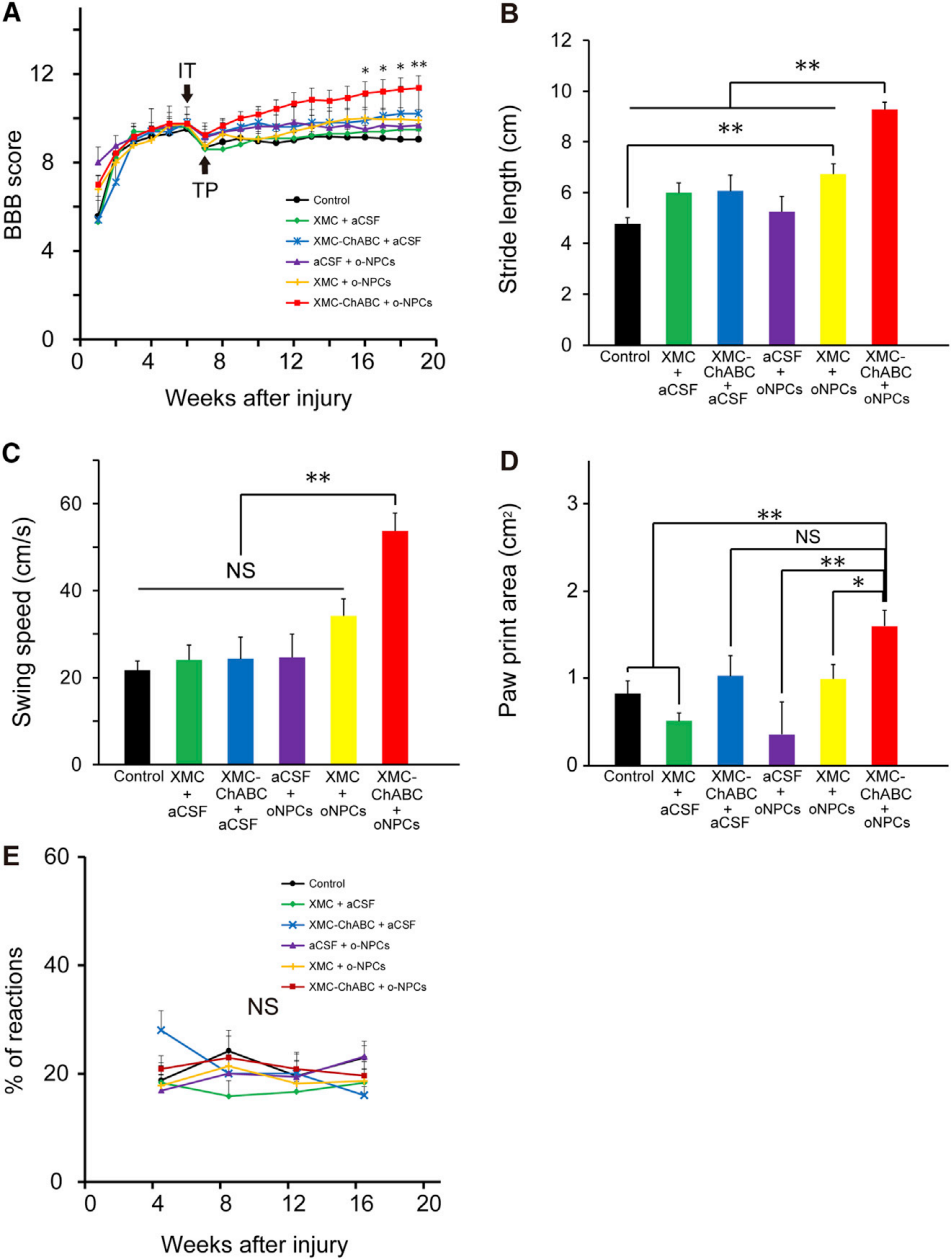
XMC-ChABC and oNPCs Combinatorial Therapy Improves Motor Function after Chronic SCI and Does Not Exacerbate Neuropathic Pain (A) Hindlimb motor function was assessed weekly using the BBB score for 19 weeks. The XMC-ChABC + oNPCs group showed significant recovery from 16 to 19 weeks after SCI (XMC-ChABC + oNPCs, n = 12; XMC-ChABC + aCSF, n = 5; XMC + oNPCs, n = 11; XMC + aCSF, n = 5; aCSF + oNPCs, n = 8; control, n = 12). (B–D) Gait analysis was performed using the CatWalk system at 18 weeks after SCI (XMC-ChABC + oNPCs, n = 12; XMC-ChABC + aCSF, n = 4; XMC + oNPCs, n = 10; XMC + aCSF, n = 5; aCSF + oNPCs, n = 8; control, n = 9). (B) Stride length, (C) Swing speed and (D) Paw print area. (E) Mechanical sensitivity was examined on a monthly basis using the von Frey test (XMC-ChABC + oNPCs, n = 12; XMC-ChABC + aCSF, n = 5; XMC + oNPCs, n = 11; XMC + aCSF, n = 5; aCSF + oNPCs, n = 8; control, n = 12). Values are expressed as the means ± SEM. ^∗^p < 0.05, ^∗∗^p < 0.01. NS, non-significant.

Considerable STEM121^+^/MBP^+^ double-positive human myelin sheaths were observed in the injured spinal cord, suggesting that transplanted cells differentiated into mature oligodendrocytes, subsequently forming thick myelin sheaths (Figures 3C and 3D). Immunohistochemical analyses revealed STEM121^+^/MBP^+^ human myelin sheaths surrounding host rat neurofilament 200 (NF200)^+^ axons (Figures 3E and 3F). Immunoelectron microscopy showed that remyelinating myelin lamellae were strongly associated with immunogold-labeled STEM121^+^ human cells (Figures 3G-1 and 3G-2). There were more myelin lamellae associated with STEM121^+^ cells in the XMC-ChABC + oNPCs group (Figure 3G-3) than in the aCSF + oNPCs and the XMC + oNPCs groups (Figures 3G-4 and 3G-5). These results clearly demonstrated that grafted human oNPC-derived oligodendrocytes form mature myelin sheaths on spared rat axons. Furthermore, graft-derived myelination promoted the creation of nodes of Ranvier with the expression of paranodal Caspr and the juxtaparanodal voltage-gated potassium channel Kv1.2 (Figures 3H, S2A, and S2B). There were more Kv1.2 and Caspr double-positive paranodal clusters in the XMC-ChABC + oNPCs group than in the aCSF + oNPCs and the XMC + oNPCs groups around the lesion epicenter (Figures S2C and S2D).

### Promotion of Synaptic Connectivity Anterior Horn Neurons after Chronic SCI with the Combinatorial Administration of oNPCs and XMC-ChABC

We then evaluated the degree of preserved tissue and lesion volume in the injured spinal cords via Luxol fast blue and H&E staining at 19 weeks after SCI (Figure 4). There was no significant difference in the size of the white matter, gray matter, lesion area, or cavity area among the separate groups (Figures 4B–4E). To investigate the number of preserved synapses in anterior horn neurons, we quantified the presynaptic boutons in neurons located in the anterior horn at 5–7 mm caudal to the lesion epicenter. The number of presynaptic boutons in the XMC-ChABC and oNPC combinatorial therapy group exhibited significantly more synapses in the neurons than were observed in the other groups (Figures 4F–4H). Immunostaining using anti-human-specific neuron-specific enolase and anti-synaptophysin antibody revealed that grafted human-derived neurons expressed synaptophysin (Figure S3). This was further confirmed using immunoelectron microscopy and immunogold-labeled STEM121^+^ human presynaptic and postsynaptic structures, and synapses between host rat neurons and graft-derived neurons in the XMC-ChABC + oNPCs group caudal to the lesion epicenter (Figures 4I and 4J).

### XMC-ChABC and oNPC Combinatorial Therapy Improves Motor Function after Chronic SCI and Does Not Exacerbate Neuropathic Pain

**Figure 6:**
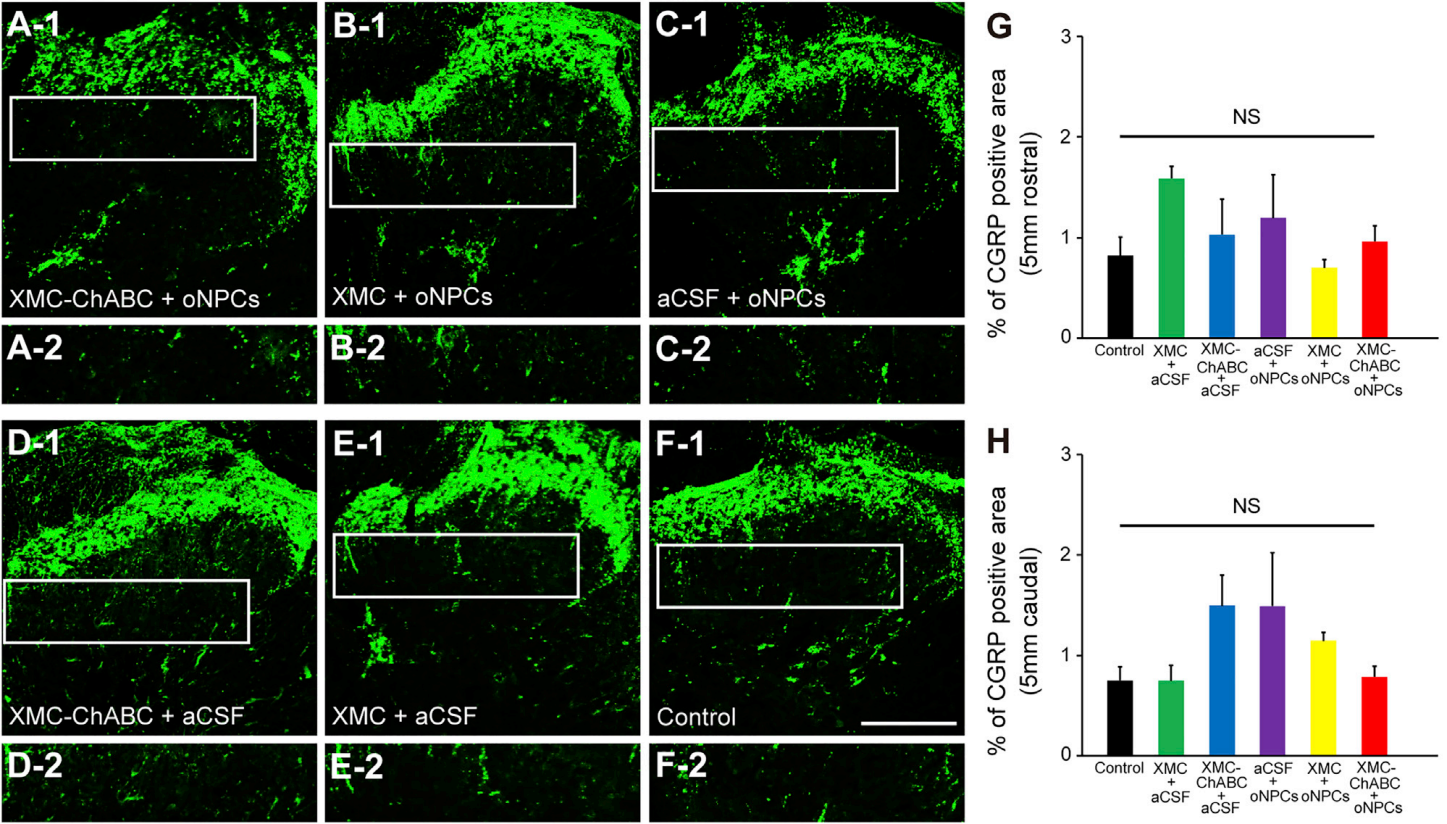
XMC-ChABC or oNPC Treatment Does Not Induce Aberrant Plasticity of Pain-Related CGRP^+^ Afferents after Chronic SCI (A–F) Representative images of CGRP^+^ fibers in the dorsal horn, 5 mm caudal to the lesion epicenter. A-2 to F-2: boxed area in A-1 to F-1, respectively. (G) CGRP^+^ area (%) in laminae III-V 5 mm rostral to the lesion epicenter at 19 weeks after SCI (n = 4/group). (H) CGRP^+^ area (%) in laminae III-V 5 mm caudal to the lesion epicenter at 19 weeks after SCI (n = 4/group). Values are expressed as the means ± SEM. NS, non-significant. Scale bar, 200 μm (F-1).

Coordinated locomotor functional recovery was assessed using the Basso, Beattie, Bresnahan (BBB) Locomotor Rating Scale and the CatWalk digital gait analysis system (Noldus, Wageningen, the Netherlands). The BBB scores indicated that only the XMC-ChABC and oNPC combinatorial therapy group exhibited significantly better functional recovery when compared with the aCSF intrathecal injection and aCSF intraspinal injection (control) group at 16 weeks post-SCI (Figure 5A). CatWalk analysis revealed that the combinatorial therapy group had significantly better recovery in terms of stride length and swing speed relative to the other groups (Figures 5B and 5C). Paw print area was also significantly larger in the combinatorial group than XMC alone, or oNPC alone (Figure 5D). We also examined mechanical sensitivity using the von Frey test, and no differences were observed between the groups (Figure 5E). Furthermore, changes in the expression of calcitonin gene-related peptide (CGRP), which is involved in peripheral and spinal pain mechanisms, were evaluated (Figures 6A–6F). We quantified the sprouts of CGRP^+^ fibers in laminae III-V at 5 mm rostral and 5 mm caudal to the lesion epicenter. There were no significant differences in the area of CGRP^+^ fibers among the groups (Figures 6G and 6H).

## Discussion

Since the glial scar is well established in the chronic stage of SCI and is known to inhibit axon regeneration as well as migration and survival of engrafted cells, a combinatorial therapy that includes degradation of the glial scar in combination with cell transplantation is likely required in order to achieve substantial functional recovery (Cregg et al., 2014). We designed a combinatorial therapy that improves grafted oNPC-mediated regeneration by degrading CSPGs using XMC-ChABC. This study was planned as a preclinical trial for chronic SCI. We used intrathecal injection of XMC-ChABC, which is less invasive than, and does not share the infection risk profile of, the typically employed osmotic mini-pump with intrathecal catheters (Pakulska et al., 2013, Pakulska et al., 2017). Furthermore, we used human directly reprogrammed oNPCs that do not involve the pluripotent state, thus making these cells potentially safer than iPSC-derived NPCs.

In this study, we observed the successful degradation of CSPGs that led to better grafted oNPC survival and migration around the lesion epicenter in the chronically injured spinal cord. XMC-ChABC-treated groups showed increased differentiation of grafted cells to oligodendrocytes and increased remyelination of the spared axons by graft-derived myelin. Moreover, the XMC-ChABC and oNPC combinatorial therapy enhanced the preservation of functional synapses in anterior horn neurons caudal to the lesion epicenter. Ultimately, the combinatorial approach resulted in significantly better motor functional recovery following chronic SCI as assessed by BBB and CatWalk outcome measures.

Previously, we developed a novel delivery strategy that provided sustained and bioactive release of ChABC to the injured spinal cord (Fuhrmann et al., 2018, Pakulska et al., 2013, Pakulska et al., 2017). ChABC, expressed as a fusion protein with SH3 (ChABC-SH3) was released from XMC covalently modified with a specific SH3-binding peptide. This allowed for the sustained release of SH3-ChABC, the bioactivity of which matches that of commercial ChABC, for at least 7 days via the affinity of SH3-ChABC to XMC-SH3-binding peptide (Pakulska et al., 2013). At both 1 and 7 days after intrathecal injection of XMC-ChABC, intact ChABC was detected at all depths within the spinal cord (Pakulska et al., 2017). Consequently, CSPG levels were observed to be decreased for at least 2 weeks in the injured spinal cord (Pakulska et al., 2017). Here, we demonstrated a clear survival advantage for oNPCs transplanted into animals pre-treated with XMC-ChABC, suggesting that the chronically injured spinal cord niche treated with XMC-ChABC is more conducive to regeneration. In addition, ChABC has been reported to enhance cell migration and distribution (Karimi-Abdolrezaee et al., 2010, Pakulska et al., 2017).

Grafted cell integration into the chronically injured spinal cord observed in this study was consistent with previous combinatorial therapies of ChABC and mouse NPCs or mouse iPSC-derived NPCs for the treatment of rodent chronic SCI (Karimi-Abdolrezaee et al., 2010, Suzuki et al., 2017). Grafted cell distribution around the lesion epicenter is important for the regeneration of the injured spinal cord, while inadequate migration of grafted cells results in poor functional recovery after SCI (Grijalva et al., 1996). Previous studies have indicated that the restricted distribution of grafted NPCs due to the glial scar results in a lack of functional recovery in chronic SCI (Nishimura et al., 2013). Nishimura et al. subsequently concluded that alteration of the microenvironment in chronic SCI, such as the degradation of the glial scar, appears to be a promising approach to maximize the therapeutic potential of NPC transplantation.

Previous research has demonstrated that stem cell maintenance is influenced by CSPGs (Purushothaman et al., 2012). Another study showed that CSPGs directly inhibit NPC properties including growth, attachment, survival, and proliferation (Dyck et al., 2015). Moreover, CSPGs preferentially differentiate NPCs to an astrocytic fate and limit their differentiation to an oligodendrogenic lineage, while CSPG receptor knockouts are seen to increase the differentiation of NPCs to oligodendrocytes (Dyck et al., 2015). XMC-ChABC treatment has proven effective in increasing the oligodendrocyte differentiation of oNPCs while decreasing astrocyte differentiation when plated in SCI-h *in vitro*. Moreover, XMC-ChABC-treated rats demonstrate greater oligodendrocyte differentiation and less astrocyte differentiation compared with non-ChABC-treated rats after oNPC transplantation in the chronically injured spinal cord. Previously, we showed that the intrathecal administration of ChABC and transplantation of NPCs biased to an oligodendrogenic fate by the infusion of a growth factor cocktail promoted neurobehavioral recovery after chronic SCI (Karimi-Abdolrezaee et al., 2010).

Past work has demonstrated remyelination of denuded host axons by graft-derived oligodendrocytes, which consequently contributes to motor functional recovery after SCI (Karimi-Abdolrezaee et al., 2010, Kawabata et al., 2016, Yasuda et al., 2011). Consistently, we observed a number of STEM121^+^/MBP^+^ graft-derived human myelin sheaths in the injured spinal cord, suggesting that graft-derived human oligodendrocytes form myelin sheaths. Furthermore, we also found large amounts of graft-derived myelin sheaths surrounding NF200^+^ axons. Immunoelectron microscopy revealed that myelin cytoplasm with immunogold-labeled STEM121^+^ spots was strongly associated with remyelinating myelin lamellae. These findings indicated that grafted human oNPC-derived oligodendrocytes formed myelin sheaths and enwrapped spared axons in the chronically injured spinal cord. Consistent with the greater oligodendrocyte differentiation in the XMC-ChABC-treated group, this group showed more oNPC-derived myelin lamellae compared with the non-ChABC-treated groups. Furthermore, Kv1.2^+^/Caspr^+^ nodes of Ranvier were observed in graft-derived myelin sheaths, suggesting that grafted human oNPC-derived myelin sheaths were functional. We also observed more paranodal clusters in the XMC-ChABC and oNPC combinatorial therapy group than in the aCSF + oNPCs and XMC + oNPCs groups. We suggest that the combinatorial therapy promoted creation of graft-derived functional myelin sheaths as well as preservation of host-derived functional myelin sheaths.

Although we observed a trend in the preservation of white and gray matter areas, and decrease in the lesion area with the combination of XMC-ChABC and oNPCs, these effects were not significant. Several previous studies demonstrated that the transplantation of NPCs or oligodendrocyte progenitor cells from different sources enhanced tissue sparing after subacute SCI (Nori et al., 2011, Yasuda et al., 2011). As we started the combinatorial therapy in the chronic phase (6 weeks after SCI), overwhelming cell death and degeneration in the acute phase of SCI had already promoted the loss of tissue volume and cystic cavity formation by the time of therapeutic intervention (Karimi-Abdolrezaee et al., 2010). Due to the chronic phase of injury, the XMC-ChABC and oNPC combinatorial therapy might not promote tissue preservation in this study.

We also quantified the presynaptic boutons in the anterior horn neurons at 5–7 mm caudal to the lesion epicenter, and more synapses were preserved in the neurons of the combinatorial therapy group. Therefore, although the combinatorial therapy did not promote tissue preservation, it promoted the preservation of functional synapses caudal to the lesion epicenter in chronic SCI. We also observed the formation of synapses between graft-derived human neurons and host rat neurons in the combinatorial therapy group. These newly formed synapses might also contribute to the overall increase in synapses observed in the neurons of the combinatorial therapy group, resulting in neurobehavioral recovery. Abematsu et al. (2010), as well as Fujimoto et al. (2012), used a wheat germ agglutinin tracer to reveal that grafted NPCs or human iPSC-derived neuroepithelial-like stem cells promoted reconstruction of the corticospinal tract in a relay fashion: the graft-derived neurons reconstructed the disrupted neuronal circuits, thereby acting as relays for transmitting signals between host-derived neurons whose interconnection had been abolished by the SCI. Using a neuronal retrograde tracer and laser microdissection, Yokota et al. (2015) revealed that reorganization of propriospinal circuits through synapse formation between graft-derived neurons and host-derived neurons directly contributed to functional recovery after NPC transplantation. In the current work, we demonstrated that combinatorial therapy promoted the preservation of functional synapses caudal to the lesion epicenter and synapse formation between graft-derived human neurons and host rat neurons. However, we did not directly show evidence demonstrating the relationship between the graft-derived neurons and motor functional recovery. Further study with a neuronal tracer is required to assess the direct contribution of oNPC-derived neurons to functional recovery.

Of note, our results demonstrated that the XMC-ChABC and oNPC combinatorial therapy did not enhance the plasticity of pain afferents in the dorsal horn of the spinal cord or increase neuropathic pain after chronic SCI. Previous studies have shown that grafted NPCs principally differentiated into astrocytes and were associated with aberrant sprouting of CGRP^+^ fibers, leading to increases in neuropathic pain (Hofstetter et al., 2005). However, previous work from our lab revealed that neuropathic pain was not observed if oligodendroglial differentiation was enhanced (Karimi-Abdolrezaee et al., 2010). In the current study, grafted cells mainly differentiated into oligodendrocytes (50.2% ± 1.4%) in the combinatorial therapy group. Therefore, we did not observe increased pain sensitivity as a result of the XMC-ChABC and oNPC combinatorial therapy, supporting the safety of this treatment.

The strengths of this study are related to the use of human drNPCs biased toward an oligodendrogenic fate and administration with ChABC through a biomaterial-based, affinity release delivery system. Furthermore, this strategy has considerable clinical relevance. Although most patients with SCI are in the chronic phase of the injury, effective therapeutic options for this population are lacking. Previously, our lab has shown that transplantation of oNPCs promotes motor functional recovery after subacute SCI (Nagoshi et al., 2018). Given that many patients are in the chronic phase of SCI, here we investigated the therapeutic impact of oNPCs for chronic injury. Due to this direct reprogramming approach that does not depend on pluripotency pathways, human directly reprogrammed oNPCs are a potential safe cell source for the treatment of SCI.

Moreover, we used an innovative methylcellulose delivery strategy that allowed sustained and bioactive release of ChABC. A single injection of XMC-ChABC is less invasive and less prone to complications, such as infection, than the osmotic mini-pump with intrathecal catheters (Follett et al., 2004, Pakulska et al., 2017). However, it needs to be emphasized that our current study is only a first step toward clinical applications. Although most patients with SCI have progressed past the subacute period and more than half of patients are severely disabled, studies focusing on chronic and severe contusion SCI are rare (Shinozaki et al., 2016). A few reports have revealed motor functional recovery in chronic and severe contusion SCI by cell transplantation (Granger et al., 2012, Zurita and Vaquero, 2006), administration of ChABC (Hu et al., 2018), combined treatment with ChABC and treadmill rehabilitation (Shinozaki et al., 2016), and intraspinal injection of biocompatible polymer hydrogel (Woerly et al., 2001). Although we observed that the combinatorial therapy of ChABC and oNPC transplantation promoted motor functional recovery in chronic and moderate contusion SCI, future studies should evaluate the efficacy of the combinatorial therapy for chronic and severe contusion SCI.

In conclusion, pretreatment of XMC-ChABC reduced CSPG levels and enhanced grafted oNPC survival, migration, and oligodendrogenic differentiation in chronic SCI. This combinatorial therapy promoted the preservation of functional synapses in the anterior horn neurons caudal to the lesion epicenter and enhanced graft-induced myelination of axons. Thus, the combinatorial therapy of XMC-ChABC and oNPCs is a clinically relevant exciting treatment option to regenerate the chronically injured spinal cord.

## Experimental Procedures

Additional details regarding several of the protocols used in this work are provided in Supplemental Experimental Procedures.

### Biasing Human drNPCs toward an Oligodendrogenic Fate

drNPCs (New World Laboratories, Laval, QC, Canada) were directly reprogrammed from human bone marrow somatic cells using defined media and transient transfection of three factors: Musashi-1, Neurogenin-2, and methyl-CpG binding domain protein 2. We generated oNPCs from drNPCs as described previously with a number of minor modifications (Khazaei et al., 2017, Nagoshi et al., 2018).

### Animals

All animal procedures were performed in accordance with the Canadian Council on Animal Care Guidelines. All experimental protocols were approved by the Animal Care Committee at the Krembil Research Institute (Toronto, Canada).

### Experimental Design and Groups

**Figure 7:**
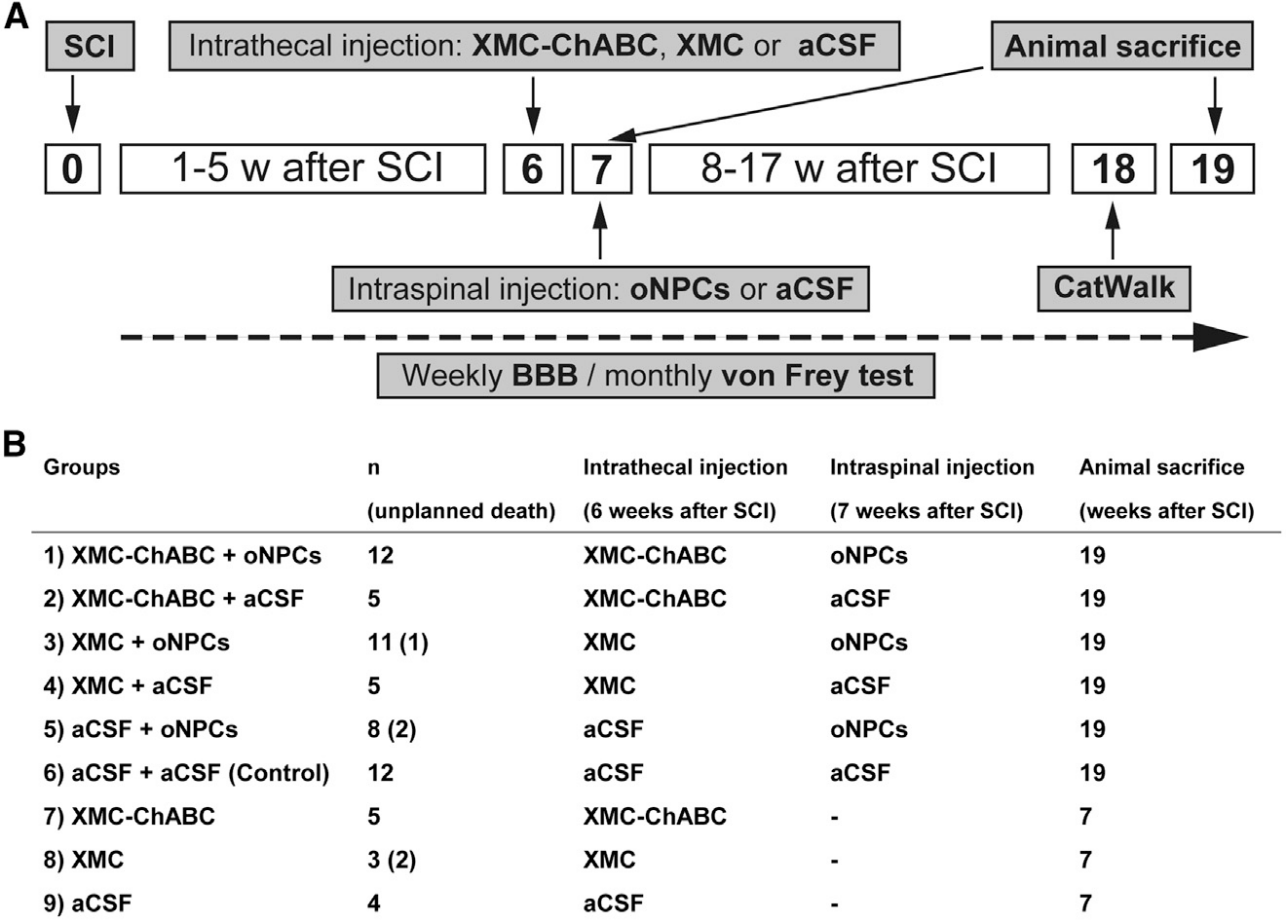
Experimental Design and Groups (A) A Th7 clip-contusion SCI was induced at week 0 in RNU rats. Six weeks after SCI, XMC-ChABC, XMC, or aCSF were intrathecally injected at the lesion epicenter. One week later, oNPCs or aCSF were injected into the cord parenchyma at two rostral and two caudal sites. BBB was performed once per week for 19 weeks. Gait analysis was performed using the CatWalk system at 18 weeks after SCI. The von Frey test was performed once per month for 16 weeks. The animals were perfused at 19 weeks (n = 53) or 7 weeks (n = 12) after SCI for immunohistochemical analyses. (B) Summary of rats used in this study. Animals in groups 1–6 were used for the histological analyses at 19 weeks after SCI. Animals in groups 7–9 were used for the histological analyses of CSPGs degradation.

Adult female RNU rats were anesthetized via inhalation. After Th7-Th9 laminectomy, a 23-g clip was applied to induce a compression injury at the Th7 level of the spinal cord. At 6 weeks after SCI, intrathecal injection was performed as described previously (Fuhrmann et al., 2018, Pakulska et al., 2017). One week after intrathecal injection (7 weeks after SCI), 4 × 10^5^ oNPCs or an equal volume of aCSF, was injected into the spinal cord. The *in vivo* experimental design is shown in Figure 7A. At 6 weeks after SCI, rats were randomized into six experimental groups based on their BBB score to ensure equivalent deficits across the groups: (1) XMC-ChABC + oNPCs, (2) XMC-ChABC + aCSF, (3) XMC + oNPCs, (4) XMC + aCSF, (5) aCSF + oNPCs, and (6) aCSF + aCSF (control) (Figure 7B). To confirm the degradation of CSPGs at 7 weeks after injury (which was the timing of cell transplantation), three other experimental groups were examined which only underwent intrathecal injection at 6 weeks: (7) XMC-ChABC, (8) XMC, and (9) aCSF (Figure 7B).

### Behavioral Assessments and Histological Analyses

All behavioral assessments were performed and analyzed by two independent examiners blinded to the experimental groups. Motor function was evaluated using the BBB open-field locomotion score and the CatWalk digital gait analysis system. Sensory function was evaluated using von Frey filaments. For histological analyses, the animals were anesthetized and transcardially perfused with 4% paraformaldehyde (pH 7.4). Spinal cords were removed and sectioned in the axial/sagittal plane on a cryostat.

### Statistical Analyses

Statistical analyses were performed using SPSS (version 22.0, IBM Corporation, Armonk, NY, USA) or Prism 6 (GraphPad Software). Mean ± SEM was used to describe continuous variables, unless stated otherwise. A one-way ANOVA, followed by a Tukey's HSD or Games-Howel's *post hoc* test for multiple comparisons, was used to analyze CS56, C4S, GFAP, survival rate of the grafted cells, *in vivo* differentiation profile, synaptic boutons, CatWalk, CGRP, and paranodal clusters analyses. Repeated-measures two-way ANOVAs, followed by Tukey's HSD tests, were used for the BBB and von Frey test analyses. Differences were considered significant at p < 0.05 (^∗^p < 0.05, ^∗∗^p < 0.01).

## Author Contributions

Conception and design: S.N., M.K., and M.G.F. Acquisition of data: S.N., M.K., C.S.A., K.Y., Y.L., J.W., S.S., and J.C. Preparation of oNPCs: M.K., J.E.A., and J.W. Preparation of the XMC: M.H.H. and T.F. Analysis and interpretation of data: S.N., M.K., K.Y., and S.S. Drafting the article: S.N., M.K., and M.G.F. Critical revision of this article: S.N., M.K., and M.G.F. Reviewed submitted version of manuscript: all authors. Approved the final version of the manuscript on behalf of all authors: M.G.F. Study supervision: M.G.F and M.S.S.

## Supplemental Information

Document S1. Supplemental Experimental Procedures, Figures S1–S3, and Table S1

Document S2. Article plus Supplemental Information
