# Supplementary material for: Human Oligodendrogenic Neural Progenitor Cells Delivered with Chondroitinase ABC Facilitate Functional Repair of Chronic Spinal Cord Injury
Source: Stem Cell Reports. 2018 Nov 21;11(6):1433–48. doi: 10.1016/j.stemcr.2018.10.017 (PMC6294173; doi:10.1016/j.stemcr.2018.10.017)
Supplement: Document S1. Supplemental Experimental Procedures, Figures S1–S3, and Table S1 [file mmc1.pdf]

Stem Cell Reports, Volume *11*

Supplemental Information

Human Oligodendrogenic Neural Progenitor Cells Delivered with Chondroitinase ABC Facilitate Functional Repair of Chronic Spinal Cord Injury

Satoshi Nori, Mohamad Khazaei, Christopher S. Ahuja, Kazuya Yokota, Jan-Eric Ahlfors, Yang Liu, Jian Wang, Shinsuke Shibata, Jonathon Chio, Marian H. Hettiaratchi, Tobias Führmann, Molly S. Shoichet, and Michael G. Fehlings

Table S1: List of Applied Biosystems™ TaqMan® probes used for qRT-PCR

ASCL1	Hs00269932
OLIG2	Hs00300164
NEUROD1	Hs01922995
NEUROG1	Hs01029249
NFIA	Hs00325656
NFIB	Hs01029174
SOX10	Hs00366918
GAPDH	Hs02786624
NKX2.2	Hs00159616
SOX9	Hs00165814
NKX6.1	Hs00232355
GFAP	Hs00909233

Supplemental Figures

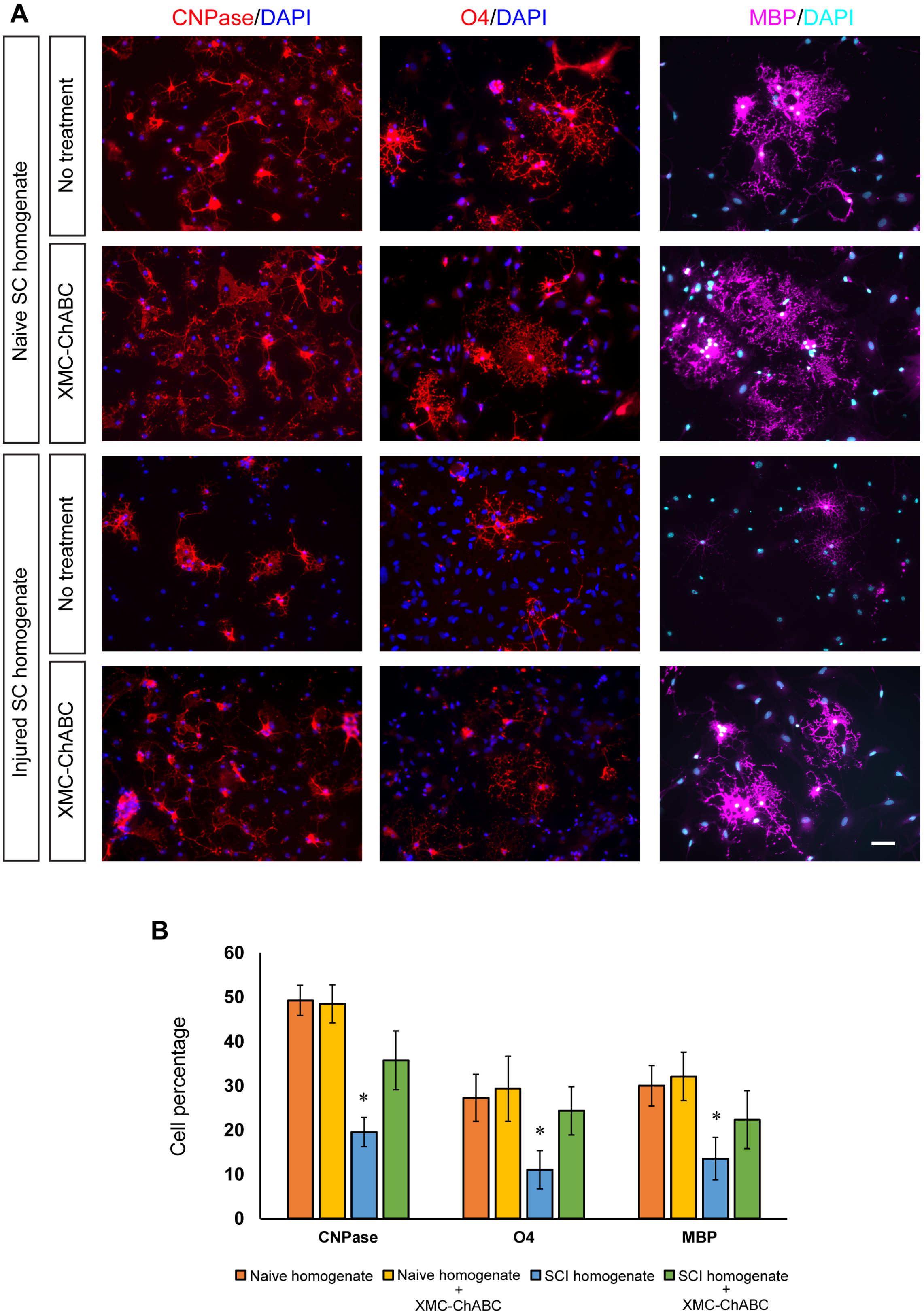

Figure S1. in vitro oligodendrogenic differentiation of oNPCs with or without CSPGs (related Figure 1).

(A) oNPCs cultured on dishes coated with spinal cord homogenate from uninjured (Naïve-h) or SCI-lesioned animals (SCI-h) were treated with or without XMC-ChABC for 1 week and were fixed and stained for oligodendrocyte markers CNPase (red). To stain for O4+ (red) and MBP+ (purple) cells were cultured in this condition for 2 weeks. (B) The percentage of cells positive for CNPase, O4, and MBP was quantified (n = 3 biological replicates/group). Values are expressed as the mean ± SD. *$p < 0.05$. (Scale bar, 20 μm).

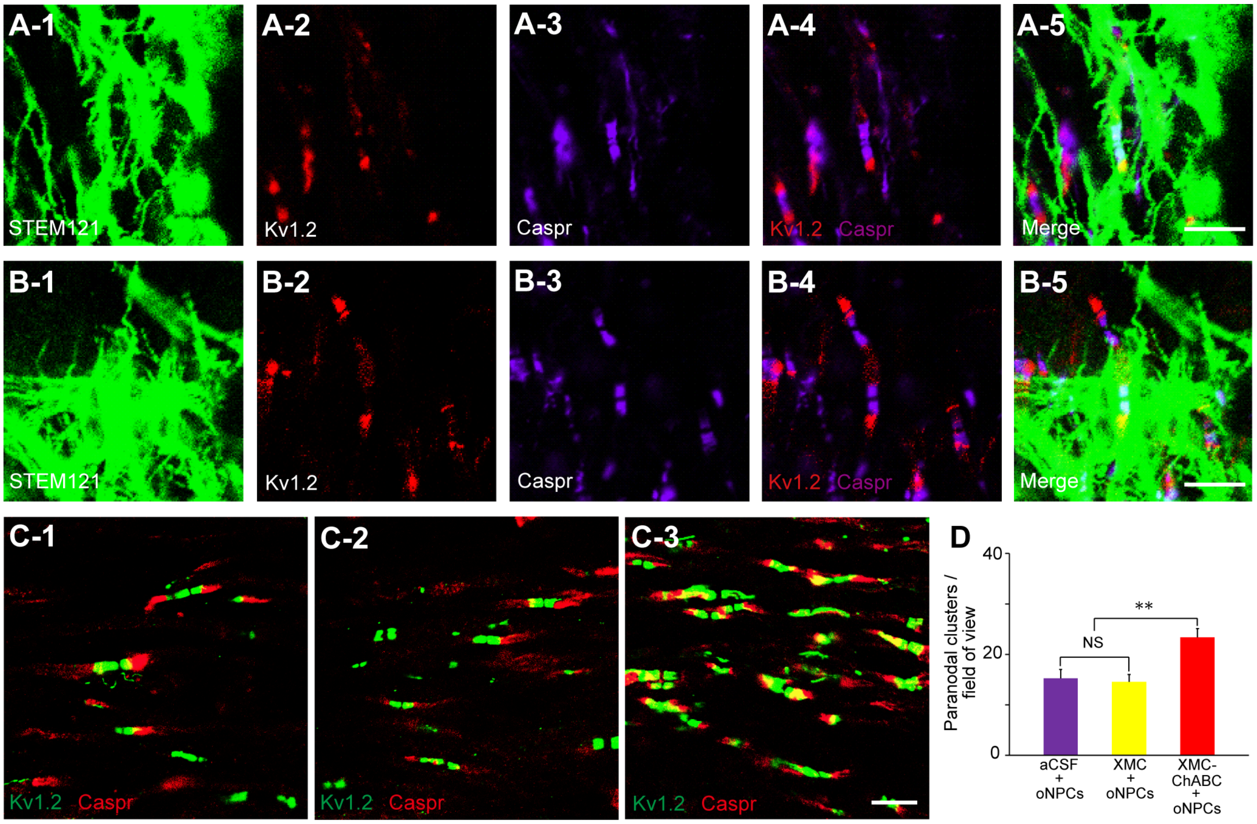

Figure S2. More paranodal clusters were observed in the combinatorial therapy group (related to Figure 3).

(A and B) Representative images of sagittal sections stained for STEM121, Kv1.2, and Caspr in aCSF + oNPCs (A) and XMC + oNPCs (B) groups. (C) Representative images of sagittal sections stained for Kv1.2+/Caspr+ paranodal clusters in aCSF + oNPCs (C-1), XMC + oNPCs (C-2) and XMC-ChABC + oNPCs (C-3) groups. (D) Quantitative analysis of the number of paranodal clusters per field of view (n = 12/group). Values are expressed as the mean ± SEM. **$p < 0.01$. NS, non-significant. (Scale bar, 5 µm in A-5 and B-5; 10µm in C-3)

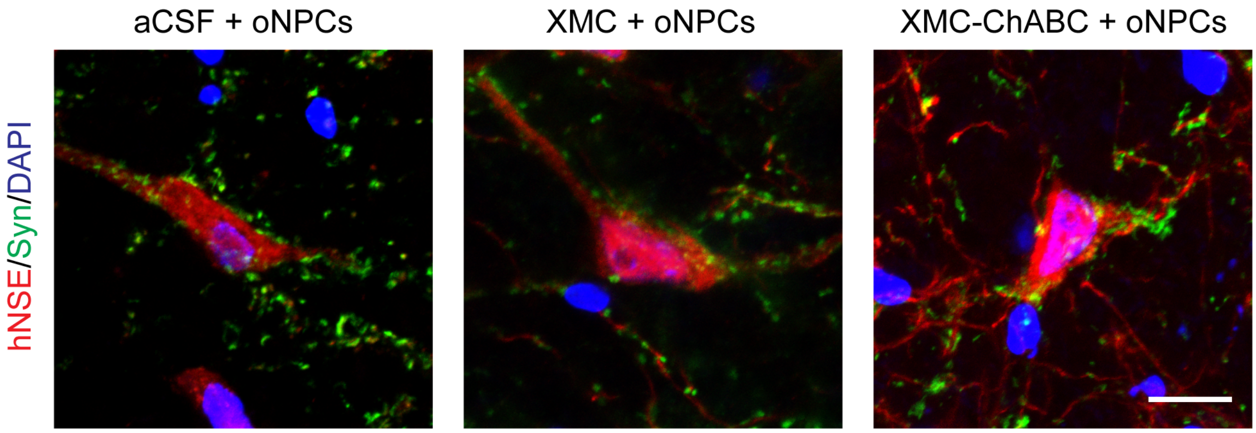

Figure S3. Grafted human-derived neurons expressed synaptophysin (related to Figure 4).

Representative images of transplanted cells stained with hNSE (red) and synaptophysin (green) (Scale bar, 10 μm).

Supplemental Experimental Procedures

Biasing human drNPCs towards an oligodendrogenic fate

The drNPCs were caudalized by culturing them in growth factor reduced matrigel in DMEM/F12, supplemented with 0.1μM retinoic acid (RA), B27 supplement (Life Technologies, Cat # 17504044), N2 supplement, and EGF (20ng/ml) for 3 days. Cells underwent ventralization by treatment with 1μM Shh agonist Purmorphamine (Millipore, Cat # 540220) for 5 days. EGF was replaced by FGF-2 (10ng/ml) from the media for 3 days followed by the addition of 20ng/ml PDGF-AA (Peptrotech 100-13A) for 14 days. The resulting cells were maintained on Laminin coated dishes in DMEM/F12, B27-A, N1 supplement (Sigma Cat # N6530), PDGF-AA (20ng/ml) and FGF-2 (20ng/ml) for 3 more passages prior to transplantation. During passaging, 10μM Rock inhibitor (Y-27632) was added on day 1.

Preparation of XMC for ChABC-SH3 delivery

XMC was prepared as described previously (Pakulska et al., 2015). Briefly, thiolated methylcellulose (Vulic and Shoichet, 2012), methylcellulose modified with SH3 binding peptides, and unmodified methylcellulose (300kDa, Shin-Etsu Corp.) were dissolved in aCSF (149 mM NaCl, 3mM KCl, 0.8 mM $MgCl_2$, 1.4 mM $CaCl_2$, 1.5 mM Na_2HPO_4, 0.2mM NaH_2PO_4) to obtain a final concentration of 5% w/v total methylcellulose and 0.1 μmol thiol/100 μl gel. The gel was crosslinked using poly(ethylene glycol)-bismaleimide ($PEGMI_2$, 3000 Da,

Rapp Polymere, Tuebingen, Germany) for a final molar ratio of 0.75:1 maleimide to thiol. Recombinant ChABC-SH3 fusion protein expressed in *E.coli*, as previously described (Pakulska et al., 2013), was added to XMC-SH3-binding peptide prior to crosslinking for a final ChABC-SH3 concentration of 0.3 U/5 μl and a final molar ratio of 1:100 ChABC-SH3:SH3 binding peptide (Fuhrmann et al., 2018; Pakulska et al., 2017).

Preparation of spinal cord homogenate

At 6 weeks after injury, rats were perfused with ice-cold phosphate-buffered saline (PBS), and a 5 mm long segment of the injured spinal cord centered on the injury epicenter was removed and rapidly frozen on dry ice (n = 5). For naïve rats, the same region of the spinal cord was removed for this preparation (n = 5) as the injured rats. Spinal cords of each group were pooled together in 1 ml DMEM:F12. The tissue was homogenized by using a set of small conical mortars and pestles for 2 min, while concurrently being kept on ice. The homogenate was cleared by centrifugation at 12000 g for 15 min, and the total protein concentration of cleared supernatants were measured using a bicinchoninic acid (BCA) test. After adjusting the total protein concentration, the aliquots were stored at −80°C until use.

***in vitro* oNPC differentiation assay**

To analyze the effect of injury induced CSPGs on the differentiation profile of oNPCs *in vitro*,

cells were cultured in SCI-h or Naïve-h coated dishes in the absence of FGF and EGF. The cells were also treated with or without XMC-ChABC (total 0.3 U/5×10^5 cells) (n = 3 biological replicates/group). After one week, cells were fixed and immunostained for anti-Nestin (rabbit IgG, 1:500, Millipore, Cat# AB5922; Burlington, MA, USA) for neural progenitor cells, anti-3CB2 (mouse IgM, 1:100, DSHB, Cat # 3CB2; Iowa, IA, USA) for radial glial cells, anti-O1 (mouse IgM, 1:500, Millipore, Cat# MAB344) for oligodendrocytes, anti-O4 (mouse IgM, 1:500, Millipore, Cat# MAB345) for oligodendrocytes, anti-CNPase (mouse IgG1, 1:500, Millipore, Cat# MAB326) for oligodendrocytes, anti-MBP (mouse IgG1, 1:500, Millipore, Cat# MAB381) for mature myelinating oligodendrocytes, anti-GFAP (mouse IgG1, 1:1000, Millipore, Cat# MAB360) for astrocytes or anti-βIII tubulin (mouse IgG2b, 1:1000; Sigma; St. Louis, MO, USA, Cat # T8660) for neurons, or used for RNA isolation and qRT-PCR.

RNA isolation and quantitative RT-PCR (qRT-PCR)

Cultured oNPCs were collected into Buffer RL (Norgen Biotek) with β-mercapthenol. Samples were processed according to the manufacturer's directions using a Total RNA Purification Kit (Norgen Biotek – Cat#17200) (n = 3 biological replicates/group). cDNA synthesis was carried out with a SuperScript® First-Strand Synthesis System for RT-PCR (ThermoFisher – Cat# 11904018). RT-PCR was carried out using a GeneAmp PCR System 9700. Cycling conditions consisted of polymerase activation and DNA denaturation (2 min at 94°C), followed by 35 cycles

of 30 s at 94°C, 30 s at 58°C, and 60 s at 72 °C. Values were normalized to the *GAPDH* housekeeping gene. See detailed information regarding primer sequences in Table S1.

Ex vivo CSPG digestion

XMC or XMC-ChABC was incubated with 200µL of PBS at 37°C. Supernatant was collected and replaced after day 1, 4, and 7. Adult female RNU rats were perfused 7 weeks after injury with 4% PFA and PBS. The injured thoracic spinal cord was dissected, immediately embedded in Shandon M1 matrix (ThermoFisher Scientific), and flash frozen. Serial axial cryosections of the lesion epicenter were mounted on slides, rinsed in PBS for 3min x 3, and dried completely. Sections were isolated with a PAP Pen (ThermoFisher Scientific) and incubated in 20µL of either PBS, stock ChABC enzyme (0.5 U/mL), or supernatant from XMC or XMC-ChABC. Slides were aspirated, rinsed with PBS 3min x 3, and labelled with anti-CS56 (mouse IgM, 1:1000, Sigma, Cat # C8035) and anti-C4S (mouse IgG1, 1:200, Millipore, Cat # MAB2030) primaries and anti-mouse IgM 568 (1:500, ThermoFisher, Cat # A-21043) and anti-mouse IgG1 647 (1:500, ThermoFisher, Cat # A-21240) secondaries with 4',6-diamidino-2-phenylindole (DAPI, 1:300, Invitrogen; Carlsbad, CA, Cat # D1306) counterstaining.

Thoracic spinal cord injury model

A total of 70 adult female RNU rats (Crl:NIH-*Foxn*1^{rnu}, 12-week-old; strain code 316; Charles

River Laboratories, Wilmington, MA) were anesthetized via inhalation using isoflurane (1-2%) and a 1:1 mixture of O_2/N_2O. After Th7-Th9 laminectomy, a 23 g clip (Walsh, Oakville, Ontario, Canada) was applied to induce a compression injury for 1 min at the Th7 level of the spinal cord. Gel foam (Ferrosan, Denmark) was put on the spinal cord, muscles were sutured, and the surgical wound was closed. Until the return of reflexive bladder control, their bladders were manually expressed twice daily.

Intrathecal injection

Intrathecal injection was performed as described previously (Fuhrmann et al., 2018; Pakulska et al., 2017). Briefly, at 6 weeks after SCI, rats were anesthetized and the spinal cord was carefully re-exposed at the injury area. A durotomy slightly caudal to the injury site was performed using a 30 gauge bent beveled needle. 5 µl of XMC-ChABC, XMC or aCSF were intrathecally injected through a 30 gauge bent blunt-tipped needle in a rostral direction (the rate was 2.5 µl/min). Upon injection, the needle was held in place for 1 min before removal.

Cell transplantation

To prepare the cell suspension, a monolayer culture of cells completed 8 passages and were collected using Accutase. Cells were diluted in aCSF and used for cell transplantation. One week after intrathecal injection (7 weeks after SCI), rats were anesthetized and the previous operative

site was carefully re-opened. The intraspinal injection was performed using a Hamilton syringe connected to a 32 gauge metal needle and stereotaxic injector. A total volume of 8 μl of cell suspension, containing 4 x 10^5 oNPCs or an equal volume of aCSF, was injected into the dorsal spinal cord. Four injection sites were mapped, 0.6-0.8 mm right and left from the midline, and 2 mm rostrally and caudally from the lesion epicenter. We delivered 1 x 10^5 cells/2 μl or 2 μl of aCSF to each site. The injected depth was 0.8-1.0 mm and the rate was 0.6 μl/min.

Behavioral assessments

The BBB score was used to assess joint movement, stepping ability, coordination, and trunk stability. Rats were evaluated once per week for 19 weeks (XMC-ChABC + oNPCs, *n* = 12; XMC-ChABC + aCSF, *n* = 5; XMC + oNPCs, *n* = 11; XMC + aCSF, *n* = 5; aCSF + oNPCs, *n* = 8; control, *n* = 12).

Gait analysis was performed using the CatWalk system at 18 weeks after SCI (XMC-ChABC + oNPCs, *n* = 12; XMC-ChABC + aCSF, *n* = 4; XMC + oNPCs, *n* = 10; XMC + aCSF, *n* = 5; aCSF + oNPCs, *n* = 8; control, *n* = 9). The footprints of the rat crossing a glass walkway were recorded by the video camera positioned below. The values for every paw were taken, and averages between left and right paws were used for analysis. Some animals could not walk along the walkway for the analysis due to insufficient motor function (XMC-ChABC + aCSF, *n* = 1; XMC + oNPCs, *n* = 1; control, *n* = 3). Using the CatWalk program (version 10.5, Noldus Inc.),

we analyzed hindlimb stride length, hindlimb swing speed and hindlimb paw print area.

Mechanical sensitivity was examined on a monthly basis after SCI for 16 weeks (XMC-ChABC + oNPCs, *n* = 12; XMC-ChABC + aCSF, *n* = 5; XMC + oNPCs, *n* = 11; XMC + aCSF, *n* = 5; aCSF + oNPCs, *n* = 8; control, *n* = 12). The rats were placed in an enclosure with a metal mesh floor. Ten mechanical stimuli (4 g von Frey filament) applied to the lateral surface of the hind paws were evaluated. Each stimulus lasted 3 seconds and was separated by a 15 min period. The number of avoidance responses (jumping, escaping, or vocalization) were counted and recorded.

Tissue processing

The animals were deeply anesthetized with 5% inhaled isoflurane and transcardially perfused with ice cold 0.1M PBS followed by 4% paraformaldehyde (PFA; pH 7.4) at 7 weeks (*n* = 12) or 19 weeks (*n* = 53) post-SCI. Dissected spinal cords (15 mm for axial slices and 20 mm for sagittal slices) were post-fixed with 10% sucrose in 4% PFA, soaked 48 hours in 20% sucrose, embedded in optimal cutting temperature (OCT) compound (Thermo Fisher Scientific, Waltham, MA), and sectioned along the sagittal/axial plane at 20 μm on a cryostat (CM3050 S, Leica, Wetzlar, Germany).

Immunohistochemistry, LFB and H&E staining

For immunostaining, the primary antibodies and the appropriate secondary antibodies were used.

Tissue sections were stained with the following primary antibodies: anti-CS56 (mouse IgM, 1:200, Sigma, Cat # C8035) for CSPGs; anti-C4S (mouse IgG1, 1:200, Millipore, Cat # MAB2030) for CSPG degradant; anti-GFAP (rabbit IgG, 1:200, Millipore, Cat # AB5804) for astrocytes; anti-human nuclear protein antibody (HuNu, mouse IgG1, 1:200; Chemicon, Boston, MA, USA, Cat # MAB1281) for human cells; anti-O1 (mouse IgM, 1:500, Millipore, Cat # MAB344) for oligodendrocytes; anti-APC (mouse IgG2b, 1:200; Calbiochem, San Diego, CA, USA, Cat # OP80) for oligodendrocytes; anti-βIII tubulin (mouse IgG2b, 1:1000; Sigma, Cat # T8660) for neurons; anti-NeuN (rabbit IgG, 1:200, Millipore, Cat # ABN78) for neurons; anti-human cytoplasm (STEM 121, mouse IgG1, 1:200, Takara Bio; Kusatsu, Japan, Cat # Y40410) for human cells; anti-MBP (rat IgG, 1:200, Abcam; Cambridge, UK, Cat # ab7349) for myelin in tissue sections; anti-NF200 (rabbit IgG, 1:200, Sigma, Cat # N4142) for axons; anti-Kv1.2 (mouse IgG2b, 1:200, Neuromab; Davis, CA, USA, Cat # P16389) for juxtaparanodal voltage-gated potassium channel; anti-Caspr (rabbit IgG, 1:200, Abcam, Cat # ab34151) for paranodal protein; anti-synaptophysin (mouse IgG1, 1:200, Abcam, Cat # AB8049) for presynaptic boutons; anti-human synaptophysin (rabbit IgG, 1:1000, Abcam, Cat # MAB324) for neurons and anti-CGRP (rabbit IgG, 1:200, Enzo Life Sciences; New York, NY, USA, Cat # CA1134) for pain-related afferents. The nuclei were stained with DAPI (1:300; Invitrogen, Cat # D1306). LFB and H&E staining was performed as previously reported (Nguyen et al., 2012). The samples were examined on a confocal laser-scanning microscope (LSM 510, Carl Zeiss,

Munch, Germany), an inverted confocal microscope (Eclipse Ti+, Nikon, Tokyo, Japan) and an epifluorescence microscope (DMR, Leica).

Immunoelectron microscopy

The detailed immunoelectron microscopy procedure has been described previously (Shibata et al., 2015). Briefly, spinal cord frozen sections with 20 mm thickness from four groups (XMC-ChABC + oNPCs, XMC + oNPCs, aCSF + oNPCs, and control) were prepared with cryostat (*n* = 2/group). Sections were incubated with the blocking solution and 5% block ace (DS Pharma Biomedical, Osaka, Japan) with 0.01% Saponin in 0.1M phosphate buffer for an hour, and stained with primary STEM 121 antibody (mouse IgG1, 1:200, Takara Bio, Cat # Y40410) for 72 hours at 4°C, followed by the incubation with nanogold conjugated secondary antibody (goat IgG, 1:100, Invitrogen, Cat # N-24915) for 24 hours at 4°C. After 2.5% glutaraldehyde fixation, nanogold signals were enhanced with R-Gent SE-EM Silver Enhancement Reagents (Aurion, Wageningen, Netherlands) for 30 minutes. Sections were post-fixed with 1.0 % OsO4 for 90 minutes at 25°C, dehydrated through graded series of ethanol and embedded in Epon. Ultrathin sections (70 nm) were prepared with ultramicrotome (UC7, Leica) and stained with uranyl acetate and lead citrate. The sections were observed under a transmission electron microscope (JEOL model 1400 plus, JEOL, Peabody, MA, USA).

Quantitative analyses of stained tissue sections

We selected two mid-sagittal sections for each rat (*n* = 6-10/group), and each section was captured using Tiling and Stitching Software from Stereo Investigator (MBF Bioscience) at 20× primary objective on an epifluorescence microscope (DMR, Leica). Using Image J Software (NIH; Bethesda, MD, USA), stained images were batch converted to 8-bit greyscale using the same threshold for specific signals between groups. After setting the threshold, $CSPG^+$, $C4S^+$ and $GFAP^+$ area was measured. For each of these antibodies stained area was normalized to the total area of the spinal cord sagittal section.

The number of surviving grafted oNPCs was estimated by staining with DAPI and anti-HuNu on serial spinal cord sections 0.5 mm apart using the optical fractionator method (*n* = 7/group) (West et al., 1991). Unbiased stereological techniques with an optical fractionator probe from Stereo Investigator (MBF Bioscience, Williston, VT) on an epifluorescence microscope (DMR, Leica) were used. Under 20× primary objective, the counting procedure began by drawing contour lines with the stereologic software in order to isolate the areas with $HuNu^+$ cells on each axial section. Stereo Investigator software was used to produce contour lines which defined the counting area, and locations were randomly selected from this area. The size of the x-y sampling grid was 250 μm. The counting frame area was 14400 μm^2 and the counting frame thickness was 10 μm. Random counting frames were imaged under 40× primary objective.

To quantify the proportion of each cellular phenotype *in vivo*, the samples were examined using

a confocal laser-scanning microscope (LSM510, Carl Zeiss). We randomly selected and captured ten regions within 3 mm rostral and caudal to the lesion epicenter at 63× primary objective. $HuNu^+$-engrafted cells and phenotypic marker-positive cells were counted in each section (n = 5/group).

For lesion morphometry, axial serial LFB and H&E stained sections were used. A blinded investigator performed LFB and H&E analyses on tissue ±2 mm the epicenter (n = 5/group). Unbiased area measurements were performed with a Cavalieri volume probe from Stereo Investigator (MBF Bioscience) to quantify the total spinal cord, gray matter, cavity and total lesion. The lesion area was identified as having eosinophilic scar deposition with immune infiltrates and/or non-viable or anuclear host tissue (Wilcox et al., 2014). For white matter area quantification, gray matter area and lesion area were subtracted from the total spinal cord area. Images were captured for tissue sections every 0.25 mm on an epifluorescence microscope (DMR, Leica) at 20× primary objective.

To quantify the presynaptic boutons in neurons in the anterior horn 5-7 mm caudal to the lesion epicenter, neurons larger than 20 μm were captured at 60× primary objective using an inverted confocal microscope (Eclipse Ti+, Nikon). Counting the number of synaptic boutons was completed using a previously described algorithm (Ashrafi et al., 2014; Tsai et al., 2012; Yokota et al., 2015). Ten images of different neurons were obtained from five rats per group, resulting in 50 total images from each group. The average number of synaptic boutons of neurons in each

group was presented in bar graph.

To quantify the $CGRP^+$ primary afferents terminating in the dorsal horn of the spinal cord, bilateral laminae III-V areas were captured at 20× primary objective using an inverted confocal microscope (Eclipse Ti+, Nikon) at 5 mm rostral and caudal to the lesion epicenter (n = 4/group). Each image was acquired using identical laser power, gain and offset values. Using Image J Software (NIH), CGRP stained images were batch converted to 8-bit greyscale using the same threshold for specific signal between groups. We measured the area of CGRP in laminae III-V of bilateral dorsal horns, and percent $CGRP^+$ area was defined as the $CGRP^+$ area divided by the total area analyzed.

A previously described algorithm was used to quantify the number of Kv1.2 and Caspr double-positive paranodal clusters (Takano et al., 2012). We randomly selected and captured twelve regions within 1 mm rostral and caudal to the lesion epicenter at 60× primary objective using an inverted confocal microscope (Eclipse Ti+, Nikon). The average number of paranodal clusters per field of view (100 x 100 μm^2) in each group was analyzed.

References

Ashrafi, S., Betley, J.N., Comer, J.D., Brenner-Morton, S., Bar, V., Shimoda, Y., Watanabe, K., Peles, E., Jessell, T.M., and Kaltschmidt, J.A. (2014). Neuronal Ig/Caspr recognition promotes the formation of axoaxonic synapses in mouse spinal cord. Neuron. *81*, 120-129.

Fuhrmann, T., Anandakumaran, P.N., Payne, S.L., Pakulska, M.M., Varga, B.V., Nagy, A., Tator, C., and Shoichet, M.S. (2018). Combined delivery of chondroitinase ABC and human induced pluripotent stem cell-derived neuroepithelial cells promote tissue repair in an animal model of spinal cord injury. Biomedical materials (Bristol, England). *13*, 024103.

Nguyen, D.H., Cho, N., Satkunendrarajah, K., Austin, J.W., Wang, J., and Fehlings, M.G. (2012). Immunoglobulin G (IgG) attenuates neuroinflammation and improves neurobehavioral recovery after cervical spinal cord injury. Journal of neuroinflammation. *9*, 224.

Pakulska, M.M., Tator, C.H., and Shoichet, M.S. (2017). Local delivery of chondroitinase ABC with or without stromal cell-derived factor 1alpha promotes functional repair in the injured rat spinal cord. Biomaterials. *134*, 13-21.

Shibata, S., Murota, Y., Nishimoto, Y., Yoshimura, M., Nagai, T., Okano, H., and Siomi, M.C. (2015). Immuno-Electron Microscopy and Electron Microscopic In Situ Hybridization for Visualizing piRNA Biogenesis Bodies in Drosophila Ovaries. Methods Mol Biol. *1328*, 163-178.

Takano, M., Hikishima, K., Fujiyoshi, K., Shibata, S., Yasuda, A., Konomi, T., Hayashi, A., Baba, H., Honke, K., Toyama, Y., et al. (2012). MRI characterization of paranodal junction failure and related spinal cord changes in mice. PLoS One. *7*, e52904.

Tsai, H.H., Li, H., Fuentealba, L.C., Molofsky, A.V., Taveira-Marques, R., Zhuang, H., Tenney, A., Murnen, A.T., Fancy, S.P., Merkle, F., et al. (2012). Regional astrocyte allocation regulates CNS synaptogenesis and repair. Science. *337*, 358-362.

West, M.J., Slomianka, L., and Gundersen, H.J. (1991). Unbiased stereological estimation of the total number of neurons in thesubdivisions of the rat hippocampus using the optical fractionator. Anat. Rec. *231*, 482-497.

Wilcox, J.T., Satkunendrarajah, K., Zuccato, J.A., Nassiri, F., and Fehlings, M.G. (2014). Neural precursor cell transplantation enhances functional recovery and reduces astrogliosis in bilateral compressive/contusive cervical spinal cord injury. Stem cells translational medicine. *3*, 1148-1159.

Yokota, K., Kobayakawa, K., Kubota, K., Miyawaki, A., Okano, H., Ohkawa, Y., Iwamoto, Y., and Okada, S. (2015). Engrafted Neural Stem/Progenitor Cells Promote Functional Recovery through Synapse Reorganization with Spared Host Neurons after Spinal Cord Injury. Stem Cell Reports. *5*, 264-277.
